# Low-Molecular-Weight Polyols as Key Factors in Sulfur- and Borate-Mediated Protomembrane Formation Before the RNA World

**DOI:** 10.3390/membranes16080272

**Published:** 2026-08-15

**Authors:** Valery M. Dembitsky

**Affiliations:** N.D. Zelinsky Institute of Organic Chemistry, Russian Academy of Sciences, 47 Leninsky Avenue, Moscow 111991, Russia; valeryde@ioc.ac.ru

**Keywords:** origin of life, protomembranes, proto-lipids, low-molecular-weight polyols, borate, sulfur, hydrogels, glycolipids, pH-dependent evolution, pre-phosphate earth, membrane self-assembly, protocells

## Abstract

The emergence of biological membranes was a critical step in the origin of cellular life because compartmentalization enabled molecular concentration, selective interactions, and increasingly complex chemical evolution. While fatty acids are widely considered the primary constituents of primitive membranes, the origin of the hydrophilic molecular scaffolds required for more stable amphiphilic systems remains unresolved. In this review, we propose a new conceptual framework in which low-molecular-weight polyols—including ethylene glycol, glycerol, tetritols, and related sugar alcohols—served as key molecular intermediates linking abiotic carbohydrate chemistry with the emergence of proto-lipids and protomembranes during a pre-phosphate stage of Earth history. Experimental and theoretical studies indicate that abiotic carbon chemistry can generate abundant polyols capable of esterification, etherification, hydrogen bonding, and reversible complexation with borate species. We hypothesize that borate-mediated stabilization of sugars and polyols promoted molecular selection, while sulfur-rich geochemical environments supplied chemically diverse amphiphiles and redox-active reaction networks. Building upon these observations, we propose a pH-dependent evolutionary model in which acidic sulfur-rich environments favored sulfo-protolipids, near-neutral environments promoted mixed polyol–fatty acid membranes, and alkaline boron-rich systems facilitated borate-associated amphiphiles and dynamic supramolecular membrane organization. We further suggest that borate-cross-linked polyol hydrogels acted as transitional soft-matter systems connecting molecular synthesis, membrane self-assembly, compartmentalization, and the emergence of proto-informational assemblies. Modern glycolipids, sulfolipids, archaeal ether lipids, and calditol-containing tetraether membranes are discussed as structural analogues, rather than direct evolutionary descendants, supporting the chemical versatility of polyol-based membrane architectures. Although the proposed evolutionary framework remains hypothetical, it integrates current knowledge from prebiotic organic chemistry, membrane biophysics, boron coordination chemistry, sulfur geochemistry, and systems chemistry into a unified and experimentally testable model for the evolution of proto-lipids, protomembranes, and early protocellular organization.

## 1. Introduction

The emergence of cellular membranes represents one of the most critical transitions in the origin of life because compartmentalization enabled molecular concentration, selective transport, energy transduction, and the evolution of increasingly complex biochemical systems [1,2,3,4,5,6,7,8,9]. Despite major advances in origin-of-life research, the molecular pathway leading from simple prebiotic amphiphiles to stable membrane-forming structures remains incompletely understood.

Current models of protocell formation primarily emphasize the spontaneous self-assembly of fatty acids and related amphiphiles into micelles, vesicles, and other supramolecular aggregates under plausible prebiotic conditions [1,3,5,7,9,10,11,12]. Although such systems readily form membrane-like compartments, fatty acid membranes generally exhibit limited stability, high sensitivity to environmental conditions, and restricted structural diversity. These limitations suggest that additional hydrophilic molecular scaffolds may have contributed to the emergence of more robust membrane architectures during early chemical evolution [2,4,6,10].

Among the numerous classes of prebiotic organic compounds, low-molecular-weight polyols occupy a unique position [1,6,13,14,15,16,17]. Formed through abiotic carbon chemistry involving formaldehyde, glycolaldehyde, glyceraldehyde, and related intermediates, polyols are predicted to occur abundantly, with ethylene glycol, glycerol, and tetritols dominating the product distribution [1,6,14,17]. Their chemical stability, multiple hydroxyl groups, and exceptional structural versatility make them attractive candidates as hydrophilic precursors of early amphiphilic molecules.

The incorporation of polyols into amphiphiles represents a plausible evolutionary step beyond simple fatty acid membranes. Reactions between polyols and hydrophobic compounds can generate diverse neutral lipids, glycolipids, and related amphiphiles with improved self-assembly properties [1,2,3,4,6,7,17]. Such molecules could have promoted the transition from primitive amphiphilic films to increasingly stable protomembranes, providing a molecular foundation for membrane complexity before the emergence of modern phospholipid-based systems.

Another factor likely to have influenced this transition is boron chemistry [18,19]. Borate minerals selectively stabilize sugars and polyols through reversible complexation with cis-vicinal diol groups, thereby influencing their stability, persistence, and distribution in prebiotic environments [18,19,20,21,22,23]. These interactions may have promoted the accumulation of polyol-rich molecular pools while facilitating the formation of increasingly complex amphiphilic structures during a pre-phosphate stage of chemical evolution [1,6,17].

Although previous studies have investigated fatty acid vesicles, borate stabilization of carbohydrates, archaeal ether lipids, and prebiotic membrane models separately, the potential role of low-molecular-weight polyols as molecular bridges linking carbohydrate chemistry with membrane evolution has received comparatively little attention. In this review, we propose a conceptual evolutionary framework in which polyols occupy a central position between abiotic carbohydrate synthesis and the emergence of proto-lipids and protomembranes. We examine their abiotic formation, predicted distribution, interactions with borate species, incorporation into amphiphilic molecules, and possible contribution to membrane self-assembly during the pre-phosphate era. We further distinguish experimentally supported observations from the proposed evolutionary hypothesis and identify experimentally testable pathways for evaluating the role of polyols in early protocellular evolution.

## 2. The Pre-Phosphate Earth

The chemical environment of the early Earth differed profoundly from that of the modern biosphere [24,25,26,27]. Before phosphate became the dominant component of biological membranes and nucleic acids, the Hadean and early Archean Earth was characterized by intense volcanic activity, widespread hydrothermal circulation, reducing to weakly oxidizing conditions, and a diverse inventory of inorganic compounds capable of participating in prebiotic chemistry [28,29,30]. During this period, the availability, reactivity, and environmental distribution of chemical elements likely influenced the direction of molecular evolution.

Although phosphorus is indispensable in modern biology, its role during the earliest stages of chemical evolution remains uncertain because its bioavailability was probably limited. Most phosphorus was likely locked within relatively insoluble phosphate minerals, restricting its participation in aqueous prebiotic reactions [31,32,33]. Consequently, several authors have proposed that early molecular evolution relied initially on alternative chemical systems before phosphate became incorporated into biological structures and metabolic pathways [34,35,36,37].

Among the elements that may have played important roles during this pre-phosphate stage, sulfur and boron are particularly significant [35,36,37]. Sulfur-containing minerals and hydrothermal fluids were abundant on the Hadean and early Archean Earth. Hydrogen sulfide, elemental sulfur, polysulfides, thiosulfates, and metal sulfides participated in redox transformations, catalyzed organic reactions, and generated chemically active environments favorable for the synthesis and modification of organic compounds [38,39,40,41,42,43,44]. Sulfur-rich hydrothermal systems have therefore been widely regarded as plausible settings for early chemical evolution.

Boron may have played a complementary role. Boron-bearing minerals likely occurred in localized evaporitic, volcanic, and hydrothermal environments [45,46,47]. Unlike most inorganic elements, borate exhibits a strong and selective affinity for cis-vicinal diol groups present in sugars and polyols. Reversible borate complexation stabilizes these compounds in aqueous solution and can influence both their persistence and their participation in ongoing reaction networks [18,19,20,21,22,23,48,49,50,51]. Experimental studies have demonstrated that borate protects carbohydrates from degradation and alters the distribution of products formed during prebiotic synthesis, suggesting that boron may have acted as a molecular selection factor during early chemical evolution [50,51,52,53,54].

We propose that the interaction between sulfur-rich geochemistry and borate-mediated molecular stabilization created a distinctive chemical environment favorable for the accumulation of polyhydroxylated compounds. Within such environments, formaldehyde, glycolaldehyde, glyceraldehyde, sugars, and low-molecular-weight polyols could have been synthesized, stabilized, and concentrated sufficiently to participate in the formation of increasingly complex amphiphilic molecules and membrane precursors [1,6,17].

In this conceptual framework, the pre-phosphate Earth represents a transitional stage of chemical evolution rather than a fully developed biochemical world. We hypothesize that this stage was characterized by dynamic interactions among simple organic molecules, polyols, amphiphiles, sulfur-mediated redox chemistry, and borate-stabilized molecular networks. The later incorporation of phosphate into membrane lipids and nucleic acids is therefore viewed as a subsequent evolutionary innovation built upon pre-existing molecular systems that had already undergone extensive chemical selection and supramolecular organization [1,6].

Understanding the physicochemical processes operating during this proposed pre-phosphate stage is essential for reconstructing plausible evolutionary pathways from simple organic molecules to membrane-bound protocellular systems. In particular, the generation, stabilization, and accumulation of low-molecular-weight polyols may represent a previously underappreciated molecular link between abiotic carbon chemistry and the emergence of the first stable protomembranes.

### The Phosphorus Availability Problem in Prebiotic Evolution

Phosphorus is an indispensable component of modern biological systems, forming the structural backbone of nucleic acids, phospholipids, and numerous metabolic intermediates [55,56]. Nevertheless, its role during the earliest stages of chemical evolution remains a subject of considerable debate. The central question is not whether phosphorus existed on the primitive Earth, but whether it was sufficiently available and chemically reactive to participate extensively in prebiotic processes [55,56,57,58].

Geochemical evidence indicates that phosphorus was present from the earliest stages of Earth’s history, primarily within phosphate-bearing minerals and meteoritic materials [31,32,33]. However, the limited solubility of most phosphate minerals probably restricted the concentration of bioavailable phosphorus in aqueous environments. In addition, phosphate activation presents a significant chemical challenge because the formation of phosphodiester bonds generally requires energetic activation or specific catalytic conditions [31,32,56,58].

In contrast, sulfur and boron possessed physicochemical properties that made them more immediately accessible to prebiotic chemistry. Sulfur species—including hydrogen sulfide, elemental sulfur, sulfides, sulfites, thiosulfates, sulfates, and polysulfides—were abundant in volcanic and hydrothermal environments and participated in diverse redox transformations [34,35,36,37,38]. Likewise, boron-bearing minerals and dissolved borate species readily interacted with sugars and polyols through reversible complexation with cis-vicinal diols, influencing molecular stability, persistence, and chemical selection [46,47].

These differences suggest that sulfur- and boron-mediated chemistry may have played disproportionately important roles during the earliest stages of molecular evolution, despite the presence of phosphorus on the primitive Earth [59,60,61]. Sulfur provided chemically diverse redox-active environments capable of driving organic transformations, whereas borate selectively stabilized carbohydrates and polyols, potentially enriching molecular species favorable for subsequent chemical evolution [1,6]. In contrast, phosphate may have become a dominant biochemical component only after suitable geological and environmental conditions increased its availability and facilitated its incorporation into increasingly complex molecular systems [62,63,64,65].

Within this context, the term “pre-phosphate era” does not imply the absence of phosphorus from the early Earth [66]. Rather, it describes a hypothetical stage of chemical evolution during which sulfur- and boron-mediated processes exerted a greater influence on molecular organization, selection, and self-assembly than phosphate-dependent chemistry. We therefore propose that the emergence of phosphate-based nucleic acids and phospholipid membranes represents a later evolutionary transition built upon pre-existing chemical systems in which sulfur chemistry promoted molecular diversification and borate chemistry promoted molecular stabilization and selection [62,63,64,65,66].

## 3. Abiotic Formation of Sugars and Polyols

The emergence of membrane-forming systems on the prebiotic Earth required not only amphiphilic molecules but also hydrophilic structural components capable of supporting increasingly complex lipid architectures [1,6,17]. Low-molecular-weight polyols are among the most plausible candidates because of their chemical stability, multiple hydroxyl groups, and central position in both modern biochemistry and proposed prebiotic reaction networks [1,67,68,69,70]. Understanding their abiotic origin is therefore essential for reconstructing plausible evolutionary pathways leading to proto-lipid and protomembrane formation.

One of the best-studied mechanisms for the abiotic synthesis of carbohydrates is the Butlerov formose reaction [71,72]. Under alkaline aqueous conditions, formaldehyde undergoes a sequence of condensation, isomerization, and rearrangement reactions that generate increasingly complex carbon skeletons. Glycolaldehyde, the first stable intermediate, subsequently participates in autocatalytic aldol reactions leading to glyceraldehyde, tetroses, pentoses, hexoses, and numerous related carbohydrates [1,6,17]. This reaction network is capable of producing chemically diverse sugar mixtures under conditions considered plausible for the primitive Earth.

Glyceraldehyde occupies a particularly important position within this network. As the simplest chiral carbohydrate, it provides a direct chemical link between simple carbon compounds and higher sugars [72,73,74,75,76]. Parallel reduction reactions convert aldehydes into their corresponding polyols, indicating that carbohydrate and polyol formation were likely interconnected processes rather than independent pathways. The overall sequence may be summarized as follows [1,2,71,73]:

**Formaldehyde → Glycolaldehyde → Glyceraldehyde → Sugars → Polyols**

**Figure 1:**
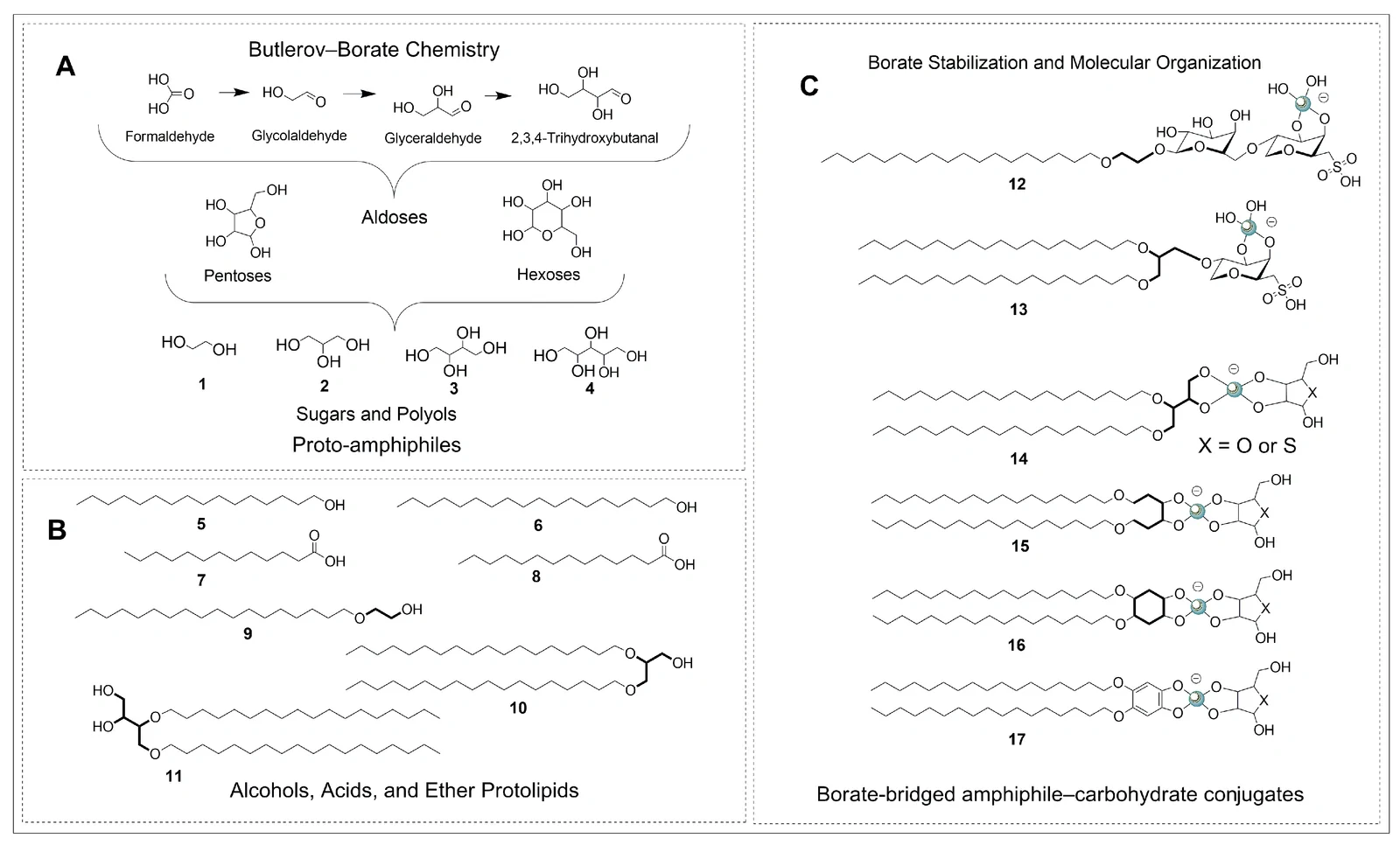
Conceptual framework for the emergence of borate-associated protolipids from prebiotic polyol chemistry. (**A**) Schematic representation of the Butlerov (formose) reaction illustrating the progressive formation of carbohydrates and low-molecular-weight polyols from formaldehyde through glycolaldehyde, glyceraldehyde, and higher aldoses. These compounds constitute the principal hydrophilic building blocks available for borate complexation and subsequent amphiphile formation. (**B**) Representative prebiotic amphiphiles, including long-chain alcohols, fatty acids, glycerol ethers, and ether-linked glycolipid precursors that may have served as early membrane-forming molecules. (**C**) Proposed borate-mediated amphiphile–carbohydrate conjugates in which reversible borate ester formation links vicinal diol-containing carbohydrates to amphiphilic molecules, generating dynamic hybrid structures with enhanced potential for molecular organization and membrane self-assembly. Collectively, these pathways illustrate a possible progression from abiotic carbohydrate synthesis to borate-stabilized protolipids during the proposed pre-phosphate stage of chemical evolution.

This pathway provides a plausible mechanism for the simultaneous accumulation of carbohydrates and sugar alcohols in prebiotic environments. Because aldehydes and sugars are susceptible to reduction under a variety of geochemical conditions, the formation of polyols may have been a common consequence of early carbon chemistry (Figure 1). Possible reducing agents include molecular hydrogen, hydrogen sulfide, transition-metal ions, mineral surfaces, and hydrothermal redox systems [77,78,79].

An important feature of this model is that the predicted distribution of polyols is highly non-random. Chemical and mathematical analyses suggest that low-molecular-weight polyols dominate the reaction products, whereas compounds containing larger numbers of hydroxyl groups become progressively less abundant [1]. According to these calculations, ethylene glycol constitutes approximately 40% of the total polyol pool, glycerol approximately 33%, and tetritols approximately 17%, together accounting for more than 90% of the predicted products [1]. Polyols containing five or more hydroxyl groups are expected to occur only in minor amounts (Table 1). This distribution probably reflects both kinetic constraints within the reaction network and the increasing tendency of larger carbohydrates to undergo cyclization and competing stabilization reactions.

The predominance of ethylene glycol, glycerol, and tetritols has important implications for membrane evolution. Their relatively small size, high aqueous solubility, multiple hydroxyl functionalities, and ability to undergo esterification and etherification make them particularly suitable as hydrophilic scaffolds for amphiphilic molecules. We therefore propose that these low-molecular-weight polyols constituted the principal molecular building blocks from which the earliest proto-lipids could have been assembled. Although this hypothesis remains to be tested experimentally, it provides a chemically plausible connection between abiotic carbohydrate synthesis and the emergence of increasingly complex membrane-forming systems.

An additional factor influencing polyol accumulation may have been borate complexation. Sugars and higher polyols readily form reversible complexes with borate ions through *cis*-vicinal diol groups [17,19,20,80,81,82,83]. Such interactions can stabilize specific molecules while simultaneously altering their participation in ongoing reaction networks. Borate-mediated molecular selection may therefore have contributed to the observed predominance of lower polyols by removing certain sugars and highly hydroxylated compounds from the reactive pool. In this manner, boron chemistry could have influenced not only carbohydrate stability but also the overall composition of prebiotic organic mixtures [1,6].

Taken together, available chemical, experimental, and theoretical evidence supports the existence of a prebiotic polyol-rich environment dominated by ethylene glycol, glycerol, and tetritols [1]. These compounds provided a readily available reservoir of multifunctional building blocks capable of participating in the synthesis of increasingly complex amphiphilic molecules. The emergence of such a polyol world represents a critical transitional stage between simple prebiotic organic chemistry and the appearance of membrane-forming proto-lipids.

## 4. Borate Stabilization and Molecular Selection

Among the inorganic elements proposed to have influenced prebiotic chemical evolution, boron occupies a unique position because of its strong and selective interaction with compounds containing cis-vicinal diol groups [17,19,20,80,81,82,83]. Unlike most inorganic ions, borate forms reversible covalent complexes with sugars, polyols, and other polyhydroxylated molecules, thereby influencing their stability, reactivity, and persistence in aqueous environments. These properties have led to the proposal that boron functioned as an important molecular selection factor during the pre-phosphate stage of Earth’s history [1,2].

Under neutral to mildly alkaline conditions, boric acid and borate ions readily react with vicinal diols to form cyclic borate esters. Such complexes are particularly favored for carbohydrates and polyols possessing appropriately oriented adjacent hydroxyl groups. Because borate ester formation is reversible, these complexes continuously assemble and dissociate in response to changes in pH, temperature, ionic strength, and reactant concentration [52,84]. This dynamic behavior distinguishes borate chemistry from the relatively stable covalent bonds characteristic of modern biological macromolecules.

One of the best-established consequences of borate complexation is the stabilization of otherwise labile carbohydrates. Experimental studies have shown that borate protects sugars against degradation and rearrangement, thereby increasing their lifetime in aqueous solution. Although ribose has received particular attention because of its role in nucleic acids, similar stabilization has been reported for numerous other sugars and polyols [52,53]. Consequently, boron-rich environments may have acted as localized reservoirs in which selected polyhydroxylated molecules accumulated preferentially [84,85,86,87].

Borate complexation may also help explain the predicted distribution of prebiotic polyols. Polyols containing four or more hydroxyl groups readily form stable borate complexes, reducing their availability for further chain extension and competing reactions. In contrast, smaller polyols such as ethylene glycol and glycerol generally exhibit weaker borate binding and therefore remain more accessible for subsequent chemical transformations. This mechanism provides a plausible explanation for the predominance of low-molecular-weight polyols predicted by theoretical and mathematical models [1].

Beyond molecular stabilization, borate may have contributed to a broader process of molecular selection [52,53,84,85,86,87]. Abiotic reaction networks likely generated highly complex mixtures containing thousands of organic compounds. Without selective processes, such chemical diversity would have hindered the emergence of organized molecular systems. Because borate preferentially recognizes specific stereochemical arrangements of vicinal diols, it selectively enriches certain carbohydrates and polyols while leaving others comparatively unstable. We therefore propose that borate acted as a primitive molecular filter that reduced chemical complexity while enriching molecular species most suitable for subsequent evolutionary processes.

The consequences of this selection may have extended beyond carbohydrate chemistry. Polyols stabilized by borate interactions would have persisted long enough to react with fatty acids, fatty alcohols, and other hydrophobic molecules, facilitating the formation of increasingly complex amphiphiles containing polyol head groups. Within the conceptual framework proposed here, borate-mediated molecular selection therefore represents a critical step linking abiotic carbohydrate chemistry with the emergence of proto-lipids and primitive membrane systems [6,17,88].

Another important feature of borate chemistry is its dynamic nature. Unlike phosphodiester bonds, borate esters form and dissociate reversibly under environmentally accessible conditions [18,19,20,21,22,23]. Such reversibility permits continuous molecular exchange, structural adaptation, and supramolecular self-organization [1,6,17,88]. Rather than producing static molecular assemblies, borate chemistry generates dynamic reaction networks capable of responding to changing environmental conditions, a property that may have been particularly advantageous during the earliest stages of chemical evolution.

Collectively, the available evidence suggests that boron performed two complementary functions during prebiotic evolution. First, it stabilized sugars and polyols, increasing their persistence in aqueous environments. Second, it selectively enriched specific polyhydroxylated compounds through reversible molecular recognition. We hypothesize that these combined effects established a chemically favorable molecular inventory from which the earliest polyol-based amphiphiles, proto-lipids, and ultimately protomembranes could emerge.

### 4.1. pH-Driven Molecular Selection on the Pre-Phosphate Earth

One of the most important, yet often underappreciated, factors influencing prebiotic chemical evolution is environmental pH [89,90]. The early Earth was chemically heterogeneous, comprising diverse geochemical settings generated by volcanic activity, hydrothermal circulation, evaporitic processes, atmospheric interactions, and mineral weathering [89,90,91,92]. Consequently, acidic, near-neutral, and alkaline environments likely coexisted, each favoring distinct reaction pathways, molecular interactions, and supramolecular assemblies.

We propose that environmental pH acted as a fundamental geochemical selector long before the emergence of biological evolution. By controlling molecular stability, solubility, reactivity, and complexation equilibria, pH would have influenced which classes of organic compounds accumulated and persisted under different environmental conditions. Rather than representing a chemically uniform planet, the prebiotic Earth likely consisted of complementary geochemical domains, each promoting distinct stages of molecular evolution.

Within this conceptual framework, acidic environments favored sulfur-rich redox chemistry, near-neutral environments promoted the self-assembly of mixed amphiphilic systems, whereas alkaline boron-rich environments enhanced borate complexation with sugars and polyols. Together, these chemically distinct settings may have generated complementary molecular inventories that collectively contributed to the emergence of proto-lipids, protomembranes, and ultimately proto-informational systems [1,2,6,17,88].

Rather than viewing these environments as isolated or sequential evolutionary stages, we suggest that they formed an interconnected planetary-scale reaction network linked by hydrothermal circulation, evaporation, precipitation, and surface water transport. Continuous exchange of molecules among these geochemical domains would have promoted molecular diversification, selection, and self-organization, thereby increasing the probability of progressively more complex prebiotic systems.

#### 4.1.1. Acidic Domain: Sulfur-Driven Chemistry

Acidic environments were probably widespread on the Hadean Earth, particularly in volcanic regions, sulfur-rich hydrothermal systems, and areas influenced by the oxidation of sulfur-containing gases. These settings were characterized by elevated concentrations of hydrogen sulfide, sulfur dioxide, elemental sulfur, sulfites, thiosulfates, sulfates, and related sulfur species [93,94]. Such compounds participated in diverse redox transformations and created chemically reactive environments capable of driving the synthesis and modification of organic molecules.

Under acidic conditions, sulfur-containing compounds are generally more stable than borate complexes. Consequently, sulfur-rich amphiphiles, thiols, thioesters, sulfolipids, and sulfur-containing carbohydrates may have accumulated preferentially in these environments [93,94,95,96]. Many of these molecules possess properties relevant to prebiotic evolution, including redox activity, hydrogen-bonding capability, amphiphilicity, and the capacity for supramolecular self-assembly.

We propose that sulfur-rich hydrothermal environments provided one of the earliest geochemical settings for molecular diversification. Sulfur chemistry may have promoted the formation of chemically dynamic reaction networks in which amphiphilic molecules, sulfur-containing carbohydrates, and thioesters interacted continuously under acidic conditions. Although direct experimental evidence remains limited, such systems represent plausible precursors of increasingly organized molecular assemblies.

Within the pH-dependent evolutionary framework proposed here, acidic domains therefore represent one of the principal geochemical environments in which sulfur-driven molecular organization could have preceded or complemented later borate-mediated molecular selection. Rather than constituting primitive genetic systems, these sulfur-rich networks may have established chemically organized reaction environments that facilitated the subsequent emergence of more complex membrane and proto-informational systems.

#### 4.1.2. Neutral Domain: Mixed Amphiphilic Chemistry

Near-neutral environments probably occupied extensive regions of the prebiotic Earth, including shallow ponds, tidal flats, coastal lagoons, and many surface hydrothermal systems. These settings favored the coexistence of diverse organic compounds and supported a broad range of condensation, hydrolysis, and self-assembly reactions [97,98]. Compared with strongly acidic or alkaline environments, neutral pH conditions provided a chemically favorable balance between molecular stability and reactivity.

Within these environments, fatty acids, fatty alcohols, polyols, sugars, and simple glycolipid-like molecules could have coexisted and interacted [1,2,6]. Such chemically heterogeneous mixtures were likely more representative of prebiotic chemistry than systems composed of a single amphiphile. Experimental studies have shown that mixed amphiphilic systems frequently exhibit greater stability, broader environmental tolerance, and improved self-assembly compared with membranes formed solely from fatty acids [1,2,3,4,7,9].

We propose that neutral environments provided optimal conditions for the gradual transition from simple fatty acid vesicles to increasingly complex polyol-containing amphiphiles. Covalent attachment of polyols to hydrophobic chains, together with extensive hydrogen bonding among hydroxyl-rich head groups, would have generated amphiphiles possessing enhanced structural diversity and improved membrane-forming properties.

Because spontaneous formation of vesicles, bilayers, and related supramolecular assemblies is favored under near-neutral conditions, these environments may have represented important sites for the emergence of mixed polyol–fatty acid proto-lipids and progressively more stable membrane architectures. Within the pH-dependent evolutionary framework proposed here, the neutral domain therefore acts as a chemical bridge linking sulfur-dominated acidic environments with borate-rich alkaline systems. Rather than representing an independent evolutionary pathway, it provided the physicochemical conditions under which molecular components originating from both domains could interact, self-assemble, and evolve toward increasingly complex protomembranes.

#### 4.1.3. Alkaline Domain: Boron-Driven Molecular Selection

Alkaline environments, particularly those associated with serpentinization-driven hydrothermal systems, alkaline lakes, and evaporitic borate deposits, may have played a unique role in prebiotic molecular evolution [99,100,101]. Under alkaline conditions, boric acid is progressively converted into tetrahedral borate species that readily form reversible complexes with cis-vicinal diol groups present in sugars, polyols, and related polyhydroxylated compounds [1,6,17,88].

Experimental studies have demonstrated that borate selectively stabilizes carbohydrates and polyols through reversible complexation, thereby increasing their persistence in aqueous solution [52,53,84,85,86]. Such interactions influence not only molecular stability but also the distribution of reaction products within complex prebiotic mixtures. We propose that borate-mediated complexation acted as a primitive molecular selection mechanism, preferentially enriching ribose, polyols, and other vicinal diol-containing molecules while reducing the effective chemical complexity of prebiotic reaction networks [6,88].

Borate coordination may also have influenced subsequent stages of molecular evolution. Stabilization of sugars and polyols increases the availability of these compounds for reactions with fatty acids, fatty alcohols, and other hydrophobic molecules, thereby creating favorable conditions for the formation of polyol-containing amphiphiles. In addition, the reversible nature of borate ester formation permits continual molecular exchange, allowing supramolecular assemblies to reorganize in response to environmental changes.

Within the conceptual framework proposed here, alkaline boron-rich environments therefore represent specialized geochemical domains in which molecular selection, rather than molecular synthesis alone, became a dominant evolutionary process. By stabilizing selected carbohydrates and polyols while promoting their incorporation into increasingly complex amphiphilic systems, borate chemistry may have facilitated the emergence of proto-lipids, protomembranes, and chemically organized proto-informational molecular networks. Although this evolutionary scenario remains hypothetical, it is consistent with the known coordination chemistry of borate and provides experimentally testable predictions concerning the role of boron in early membrane evolution.

#### 4.1.4. Integrated pH-Driven Evolutionary Model

Taken together, acidic, neutral, and alkaline environments may have functioned as complementary chemical domains operating simultaneously on the prebiotic Earth [102,103,104]. Rather than competing hypotheses, sulfur-rich and boron-rich chemistries may represent different components of a larger planetary-scale evolutionary system (Table 2).

Within this framework, pH-driven molecular selection represents a major organizing principle of prebiotic evolution. Sulfur-rich acidic environments promoted chemical diversity and redox activity, neutral environments facilitated amphiphilic self-assembly, and alkaline boron-rich environments enhanced molecular stabilization and selection [94,98,103]. The interaction of these domains may have generated the physicochemical conditions necessary for the emergence of polyol-derived proto-lipids, protomembranes, and the earliest stages of protocellular evolution.

### 4.2. Quantitative Aspects of Borate–Polyol Interactions

The role of boron in prebiotic chemistry extends beyond its qualitative ability to stabilize sugars and polyols [6,17,88]. The physicochemical properties of boric acid and borate ions provide a quantitative framework that helps explain why boron may have acted as an effective molecular selection factor during the pre-phosphate era.

In aqueous solution, boron is present primarily as boric acid, B(OH)_3_, which behaves as a weak Lewis acid rather than a classical Brønsted acid [105,106]. The equilibrium between boric acid and borate is governed by the reaction:

**B(OH)_3_ + H_2_O ⇌ B(OH)_4_^−^ + H^+^**

The pKa of boric acid is approximately 9.2 at ambient temperature, indicating that borate formation becomes increasingly favored under mildly alkaline conditions. Consequently, borate-mediated molecular stabilization is expected to be most effective in environments with pH values above approximately 8–9, such as alkaline hydrothermal systems, evaporitic basins, and serpentinization-associated aqueous environments [107,108,109].

A unique feature of borate chemistry is its strong affinity for compounds containing cis-vicinal diol groups [18,19,20,21,22,23]. These structural motifs permit the formation of cyclic borate esters in which boron adopts a tetrahedral coordination geometry. The stability of the resulting complexes depends strongly on the stereochemical arrangement of hydroxyl groups. Molecules containing properly oriented cis-diols form substantially stronger complexes than compounds lacking such configurations.

Experimental studies have shown that equilibrium association constants for borate–diol interactions typically fall within the range [81,82,83,84]:

Log K ≈ 1–6

depending on molecular structure, pH, ionic strength, and temperature. Ribose, ribitol, xylitol, mannitol, sorbitol, and related polyhydroxylated compounds often exhibit particularly favorable binding properties because they contain multiple vicinal hydroxyl arrangements capable of coordinating borate ions [14,18,19,20,110,111,112]. In contrast, simpler compounds such as ethylene glycol and glycerol generally form weaker complexes due to the presence of fewer optimal binding sites.

The preferential interaction of borate with cis-diol-containing molecules introduces an important element of molecular selection. Prebiotic reaction networks likely generated highly diverse mixtures of carbohydrates and polyols [1,6,14,17,88]. Borate complexation selectively stabilizes only a subset of these compounds, effectively reducing chemical complexity while increasing the persistence of molecules possessing favorable stereochemical arrangements. This process may have enriched specific sugars and polyols within localized boron-rich environments.

Particularly significant is the interaction between borate and ribose. Experimental studies have demonstrated that borate can stabilize ribose against degradation and rearrangement reactions more effectively than many alternative sugars [52,53,84,85,86,100]. Although ribose was only one component of complex prebiotic reaction mixtures, borate-mediated stabilization may have increased its relative abundance and persistence. Such selective stabilization has frequently been cited as a possible explanation for the eventual incorporation of ribose into RNA chemistry.

Borate complexation also influences the physicochemical behavior of amphiphilic systems. Polyol-containing amphiphiles, glycolipids, and carbohydrate-rich membrane precursors may undergo reversible borate ester formation, generating dynamic supramolecular networks [1,6,14,17,88]. These interactions can modify molecular organization, membrane stability, hydration properties, and self-assembly behavior. Because borate ester formation remains reversible under environmentally accessible conditions, such systems retain a high degree of adaptability while maintaining overall structural coherence.

The quantitative properties of borate chemistry therefore support the hypothesis that boron functioned not merely as a passive stabilizing agent but as an active molecular selector. Through pH-dependent complexation, preferential recognition of cis-vicinal diols, and stabilization of specific sugars and polyols, borate may have helped define the molecular inventory available for the emergence of proto-lipids [1,6,88], protomembranes, and early proto-informational systems during the pre-phosphate stage of chemical evolution.

## 5. Polyols as Structural Building Blocks of Proto-Lipids

The emergence of membrane-forming systems required the integration of hydrophilic and hydrophobic molecular components into stable amphiphilic structures. While fatty acids, fatty alcohols, and related hydrophobic molecules are widely regarded as plausible constituents of primitive membranes, the origin of the hydrophilic scaffolds necessary for more complex membrane architectures remains less well understood. We propose that low-molecular-weight polyols fulfilled this role during the pre-phosphate stage of chemical evolution by providing versatile molecular frameworks capable of linking carbohydrate chemistry with amphiphile formation.

Polyols possess several physicochemical properties that make them particularly suitable as proto-lipid precursors (Table 1). Their multiple hydroxyl groups provide sites for esterification, etherification, hydrogen bonding, borate complexation, and metal-ion coordination, while their high aqueous solubility facilitates interactions between hydrophilic and hydrophobic phases. Collectively, these characteristics allow polyols to function as molecular bridges connecting hydrophobic carbon chains with hydrophilic head groups.

Among the polyols predicted to dominate prebiotic environments, ethylene glycol, glycerol, and tetritols are of particular importance [1]. Chemical and mathematical models indicate that these compounds account for more than 90% of the theoretical polyol pool generated through abiotic synthesis. Their abundance would have substantially increased their probability of participating in amphiphile-forming reactions compared with less abundant higher polyols.

The simplest proto-lipids may therefore have arisen through reactions between fatty acids, fatty alcohols, and low-molecular-weight polyols [1,6,88]. Esterification of fatty acids with ethylene glycol could generate simple diol lipids, whereas reactions involving glycerol would produce glycerolipid-like structures. Tetritols and other polyols containing additional hydroxyl groups could generate increasingly diverse amphiphiles with multiple hydrophilic functionalities [1]. We suggest that these molecules represented an evolutionary advance over membranes composed solely of free fatty acids by combining hydrophobic domains with chemically versatile polyol head groups.

A notable feature of polyol-derived proto-lipids is their extraordinary structural diversity. Even relatively simple polyols can generate numerous amphiphilic architectures depending on the number, position, and type of attached hydrophobic chains (Figure 1). This diversity increases further when sugars and borate-stabilized polyols are incorporated into the reaction network. Consequently, prebiotic environments may have contained chemically heterogeneous populations of amphiphiles differing in polarity, flexibility, membrane-forming ability, and supramolecular organization.

Particularly interesting are glycolipid-like amphiphiles generated through the association of carbohydrates, polyols, and hydrophobic chains (structures 1–17, Figure 1). Modern glycolipids demonstrate that carbohydrate-rich amphiphiles readily assemble into highly ordered membrane structures. Although these modern molecules should be regarded as structural analogues rather than direct evolutionary descendants, they illustrate the remarkable capacity of polyol-containing amphiphiles to stabilize biological membranes over evolutionary timescales.

The evolutionary significance of polyol-derived proto-lipids becomes clearer when compared with simple fatty acid membranes. Fatty acid vesicles readily self-assemble under laboratory conditions but often display limited mechanical stability and marked sensitivity to pH, ionic strength, and divalent cations. Incorporation of polyol-derived amphiphiles would increase the number of hydrogen-bonding interactions while introducing more complex hydrophilic interfaces. We therefore hypothesize that mixed fatty acid–polyol membranes possessed greater structural stability, broader environmental tolerance, and enhanced potential for subsequent molecular evolution than membranes composed exclusively of fatty acids.

Modern biological membranes provide indirect support for this concept. Polyols serve as structural backbones in glycerolipids, glycolipids, archaeal ether lipids, and numerous specialized membrane lipids found throughout bacteria, archaea, plants, fungi, and animals [1,2,3]. Their widespread occurrence demonstrates that polyol-based membrane architectures are chemically robust and evolutionarily successful. Although these lipids evolved through biological pathways, they support the general principle that polyols constitute exceptionally versatile membrane-forming scaffolds.

Within the conceptual framework proposed here, proto-lipids are viewed not simply as modified fatty acids but as products of an emerging polyol-rich chemical world. The incorporation of low-molecular-weight polyols into amphiphilic molecules represents a plausible evolutionary transition linking abiotic carbohydrate chemistry with increasingly sophisticated membrane systems. These proto-lipids subsequently provided the molecular foundation for the emergence of protomembranes and compartmentalized protocellular structures.

Figure 1 summarizes the central hypothesis developed in this review. Butlerov-type chemistry is proposed to generate a diverse pool of sugars and low-molecular-weight polyols capable of reversible borate complexation under appropriate geochemical conditions [71,72]. Borate-mediated stabilization increases the persistence of vicinal diol-containing molecules while facilitating their interaction with simple amphiphiles through dynamic borate ester formation (structures 1–17). The resulting amphiphile–carbohydrate conjugates combine hydrophobic membrane-forming domains with highly hydrated polyol head groups, providing a plausible route toward increasingly organized supramolecular assemblies [1,6,17,88]. Rather than illustrating isolated chemical reactions, the three panels represent successive stages of a continuous evolutionary sequence linking abiotic organic synthesis, borate-mediated molecular selection, amphiphile formation, and the emergence of primitive membrane systems. This conceptual model provides the foundation for the following sections on borate-associated proto-lipids, protomembranes, and early protocellular evolution.

### 5.1. Modern Membrane Analogues Supporting the Polyol Hypothesis

Although the molecular composition of the earliest membranes remains unknown, modern biological membranes provide valuable structural and functional analogues that illustrate the remarkable versatility of polyol-containing amphiphiles. Numerous contemporary membrane lipids incorporate polyols, carbohydrates, and sulfur-containing head groups as essential structural elements. While these lipids are products of advanced biological evolution rather than direct remnants of prebiotic chemistry, they demonstrate that relatively simple polyhydroxylated scaffolds can support stable and functionally diverse membrane architectures. We therefore use these systems as chemical analogues rather than direct evolutionary evidence, providing insight into plausible pathways through which polyol-containing amphiphiles may have contributed to protomembrane evolution [1,6,17].

#### 5.1.1. Archaeal Tetraether Membranes

Among the most robust biological membranes known are those produced by members of the domain Archaea [2,3,113,114,115,116,117]. Many thermophilic and acidophilic archaeal species synthesize glycerol-based tetraether lipids in which two glycerol molecules are linked by long isoprenoid chains to form membrane-spanning amphiphiles. Unlike conventional phospholipid bilayers, these tetraether lipids frequently assemble into continuous membrane monolayers that exhibit exceptional resistance to high temperatures, extreme acidity, oxidative stress, and mechanical disruption [113,114,115,116,117].

Although archaeal tetraether lipids evolved through sophisticated biological pathways and should not be regarded as direct models of prebiotic membranes, they provide valuable structural analogues demonstrating that polyol-containing amphiphiles can generate exceptionally stable membrane architectures. Their physicochemical properties illustrate how polyhydroxylated head groups, combined with appropriate hydrophobic domains, produce membranes with remarkable stability under extreme environmental conditions.

We therefore use archaeal tetraether membranes as functional analogues rather than evolutionary evidence. Within the conceptual framework proposed here, they support the hypothesis that relatively simple polyol-based amphiphiles could have formed stable membrane compartments before the emergence of modern phospholipid membranes, although the molecular composition of these hypothetical protomembranes almost certainly differed from that of extant archaeal lipids.

#### 5.1.2. Calditol-Containing Membranes

Particularly relevant to the present hypothesis are calditol-containing tetraether lipids found in several thermoacidophilic archaea [118,119]. Calditol is a cyclic polyol structurally related to sugar alcohols and forms an integral component of specialized membrane lipids adapted to extreme environmental conditions. Its multiple hydroxyl groups increase hydrogen-bonding capacity, enhance membrane cohesion, and contribute to the exceptional thermal and acid stability characteristic of these archaeal membranes [118,119,120,121].

From a physicochemical perspective, calditol-containing lipids demonstrate that polyols can serve not merely as auxiliary membrane constituents but as central structural elements within highly stable membrane architectures. Although these lipids are products of biological evolution and should not be regarded as direct descendants of prebiotic amphiphiles, they provide compelling structural and functional analogues illustrating the membrane-stabilizing potential of polyol-rich head groups.

We therefore propose that calditol-containing membranes represent modern examples of a general principle rather than specific evolutionary intermediates. Their ability to stabilize membranes under extreme conditions supports the hypothesis that simpler polyol-based amphiphiles could likewise have enhanced the cohesion, durability, and environmental resilience of primitive protomembranes before the evolution of phospholipid-based cellular membranes.

#### 5.1.3. Glycolipid Membranes

Glycolipids constitute another major class of membrane amphiphiles found throughout bacteria, archaea, plants, and animals [122,123,124]. These molecules consist of carbohydrate head groups covalently linked to hydrophobic lipid chains and play important roles in membrane stabilization, molecular recognition, cell signaling, and supramolecular organization. Their widespread distribution across all domains of life underscores the versatility of carbohydrate-containing amphiphiles as membrane-forming molecules.

The structural relationship between modern glycolipids and the hypothetical carbohydrate-rich proto-lipids proposed in this review is particularly noteworthy. In both cases, hydrophobic domains are coupled to highly hydroxylated head groups capable of extensive hydrogen bonding and hydration. These physicochemical characteristics promote membrane assembly, influence interfacial properties, and provide numerous opportunities for molecular interactions.

Although modern glycolipids are products of biological evolution and should not be regarded as direct descendants of prebiotic amphiphiles, they serve as valuable structural and functional analogues illustrating the membrane-forming potential of carbohydrate-rich head groups. We therefore propose that the widespread success of glycolipid membranes reflects general physicochemical principles that may also have operated during prebiotic membrane evolution. In this context, contemporary glycolipids provide indirect support for the hypothesis that simpler polyol- and carbohydrate-containing amphiphiles could have contributed to the formation, stabilization, and progressive diversification of primitive protomembranes [1,6,14,17,88].

#### 5.1.4. Sulfolipids and Sulfur-Rich Membranes

Sulfolipids provide an additional class of modern membrane analogues linking sulfur chemistry with membrane evolution. These amphiphilic molecules contain sulfate-bearing carbohydrate head groups covalently attached to hydrophobic lipid chains and are widespread in photosynthetic organisms, bacteria, and several extremophilic microorganisms [125,126,127,128]. In contemporary biological membranes, sulfolipids contribute to membrane organization, regulation of surface charge, molecular recognition, and adaptation to environmental stress.

From a physicochemical perspective, sulfolipids demonstrate that sulfur-containing carbohydrate head groups can form stable and functionally diverse membrane architectures. Their combination of hydrophobic lipid domains with highly oxygenated sulfur-containing head groups illustrates how sulfur chemistry can be integrated into membrane structure without compromising membrane stability or biological function.

Although modern sulfolipids evolved through biological pathways and should not be regarded as direct descendants of prebiotic amphiphiles, they provide valuable structural and functional analogues for evaluating the membrane-forming potential of sulfur-containing lipids. We therefore propose that their widespread occurrence supports the broader concept that sulfur-rich amphiphiles could have represented plausible components of prebiotic membrane systems. Within the Sulfur–Boron Era framework developed in this review, sulfur-containing amphiphiles may have constituted an early stage of membrane evolution, preceding the later emergence of borate-associated amphiphiles and, ultimately, phosphate-dominated biological membranes.

#### 5.1.5. Implications for Protomembrane Evolution

Collectively, archaeal tetraether membranes, calditol-containing lipids, glycolipids, and sulfolipids demonstrate that contemporary biology employs a remarkable diversity of membrane architectures extending far beyond conventional phospholipid bilayers [118,121,123,126,128]. Despite their structural differences, these membrane systems share a common reliance on polyols, carbohydrates, and, in some cases, sulfur-containing functional groups to enhance membrane stability, intermolecular interactions, molecular recognition, and adaptation to diverse environmental conditions.

These observations should not be interpreted as evidence that modern membrane lipids are direct descendants of specific prebiotic amphiphiles. Rather, they demonstrate that polyol-rich membrane architectures are chemically feasible, structurally robust, and evolutionarily successful. The recurring use of polyhydroxylated molecular scaffolds across diverse biological membrane systems indicates that such architectures possess intrinsic physicochemical properties favorable for membrane assembly and long-term functional stability [1,6,14,17,88].

We therefore propose that these modern membrane systems provide valuable structural and functional analogues supporting the plausibility of a polyol-centered model of protomembrane evolution. Within the conceptual framework developed in this review, low-molecular-weight polyols supplied versatile hydrophilic scaffolds capable of linking hydrophobic chains, stabilizing amphiphilic assemblies through extensive hydrogen bonding and, under appropriate geochemical conditions, participating in reversible borate complexation. Together, these properties could have facilitated the emergence of increasingly stable proto-lipids and protomembranes on the pre-phosphate Earth, providing a plausible chemical bridge between simple abiotic amphiphiles and the complex membrane systems that characterize modern cellular life.

## 6. Emergence of Protomembranes

The transition from individual amphiphilic molecules to organized membrane structures represents one of the most significant steps in prebiotic evolution. While the synthesis of proto-lipids supplied the molecular components required for membrane formation, their spontaneous self-assembly into stable supramolecular architectures generated the physical compartments necessary for increasingly complex chemical systems [1,2,3,4]. We propose that this transition marked a fundamental bridge between abiotic organic chemistry and the emergence of protocellular organization.

Amphiphilic molecules possess an intrinsic tendency to minimize free energy in aqueous environments by segregating hydrophobic and hydrophilic regions. Consequently, fatty acids, fatty alcohols, glycolipids, and related amphiphiles spontaneously assemble into micelles, vesicles, bilayers, and other supramolecular structures. Numerous experimental studies have demonstrated that even relatively simple amphiphilic molecules self-assemble under environmentally plausible conditions [1,2,3,4,8,9,10,11,12,13,14,15,16,17]. These observations suggest that compartment formation may have been a natural consequence of increasing molecular complexity on the early Earth.

Primitive membranes composed primarily of fatty acids probably represented one of the earliest stages of membrane evolution. Laboratory experiments have shown that fatty acid vesicles readily form and are capable of encapsulating dissolved molecules, thereby generating localized chemical microenvironments. However, these membranes generally exhibit limited mechanical stability and considerable sensitivity to pH, ionic composition, divalent cations, and temperature [3,7,9]. Such limitations may have restricted their long-term evolutionary potential.

We propose that the incorporation of polyol-derived proto-lipids substantially expanded the structural and functional properties of these primitive membranes. Amphiphiles containing ethylene glycol, glycerol, tetritols, sugars, and related polyols introduced additional opportunities for hydrogen bonding and other intermolecular interactions within membrane assemblies [1,2,6]. These interactions could have enhanced membrane cohesion, increased structural flexibility, and generated chemically more diverse supramolecular architectures than those formed by fatty acids alone.

A particularly important consequence of polyol incorporation may have been the emergence of glycolipid-like protomembranes [1,6,14,17]. Modern glycolipids demonstrate that carbohydrate-rich amphiphiles readily assemble into highly ordered membrane structures through the combined effects of hydrophobic interactions, hydration, and extensive hydrogen-bonding networks. Although these biological membranes are not direct models of prebiotic systems, they illustrate the membrane-forming potential of polyol- and carbohydrate-rich amphiphiles. We therefore suggest that similar physicochemical principles may have operated during early membrane evolution.

Borate chemistry may have provided an additional level of membrane organization. Polyol- and sugar-containing amphiphiles are capable of forming reversible borate esters, generating dynamic supramolecular interactions within membrane assemblies [6,14,17,88]. Such reversible coordination could promote transient cross-linking, modify membrane permeability, and enhance structural cohesion while preserving membrane flexibility. Because borate ester formation is environmentally responsive, membrane organization could have continuously adapted to changes in pH, ionic strength, hydration, and borate availability.

An additional level of structural complexity may have arisen through the formation of hydrated amphiphilic networks and hydrogel-like materials. Polyol-rich molecules possess exceptional capacities for water retention and hydrogen-bond formation, facilitating the development of highly hydrated matrices capable of concentrating organic molecules [129,130,131,132,133]. We propose that these hydrogel-like systems represented transitional structures linking simple membrane assemblies with increasingly organized protocellular compartments. By concentrating reactants while maintaining dynamic exchange with the surrounding environment, they may have enhanced the efficiency of prebiotic reaction networks.

Compartmentalization itself would have provided several important evolutionary advantages. Membrane-bound microenvironments increase local concentrations of reactants, reduce dilution of unstable intermediates, facilitate the establishment of chemical gradients, and allow selective exchange with the surrounding environment [1,6,14,17]. Together, these properties create favorable conditions for increasingly complex reaction networks and represent important prerequisites for the emergence of primitive metabolic processes.

Early protomembranes were almost certainly dynamic rather than static structures. Continuous molecular exchange, growth, fusion, division, and reorganization were likely driven by fluctuations in temperature, pH, ionic composition, hydration state, and amphiphile availability [6,14,17,88]. Such environmental variability would have continually reshaped membrane assemblies, creating opportunities for molecular selection and evolutionary innovation. In this context, membrane adaptability, as well as stability, likely became an important determinant of long-term persistence.

Within the conceptual framework proposed here, the emergence of protomembranes represents considerably more than a simple self-assembly process. It marks the appearance of chemically distinct compartments capable of supporting increasingly organized molecular processes. We hypothesize that polyol-derived proto-lipids, reversible borate coordination, and amphiphilic self-assembly acted cooperatively to generate stable yet dynamic membrane systems that provided the structural foundation for subsequent protocellular evolution. These compartments may ultimately have enabled the emergence of proto-informational molecular networks, increasingly sophisticated metabolic chemistry, and, eventually, the earliest cellular life [1,2,6,14,17,88].

**Figure 2:**
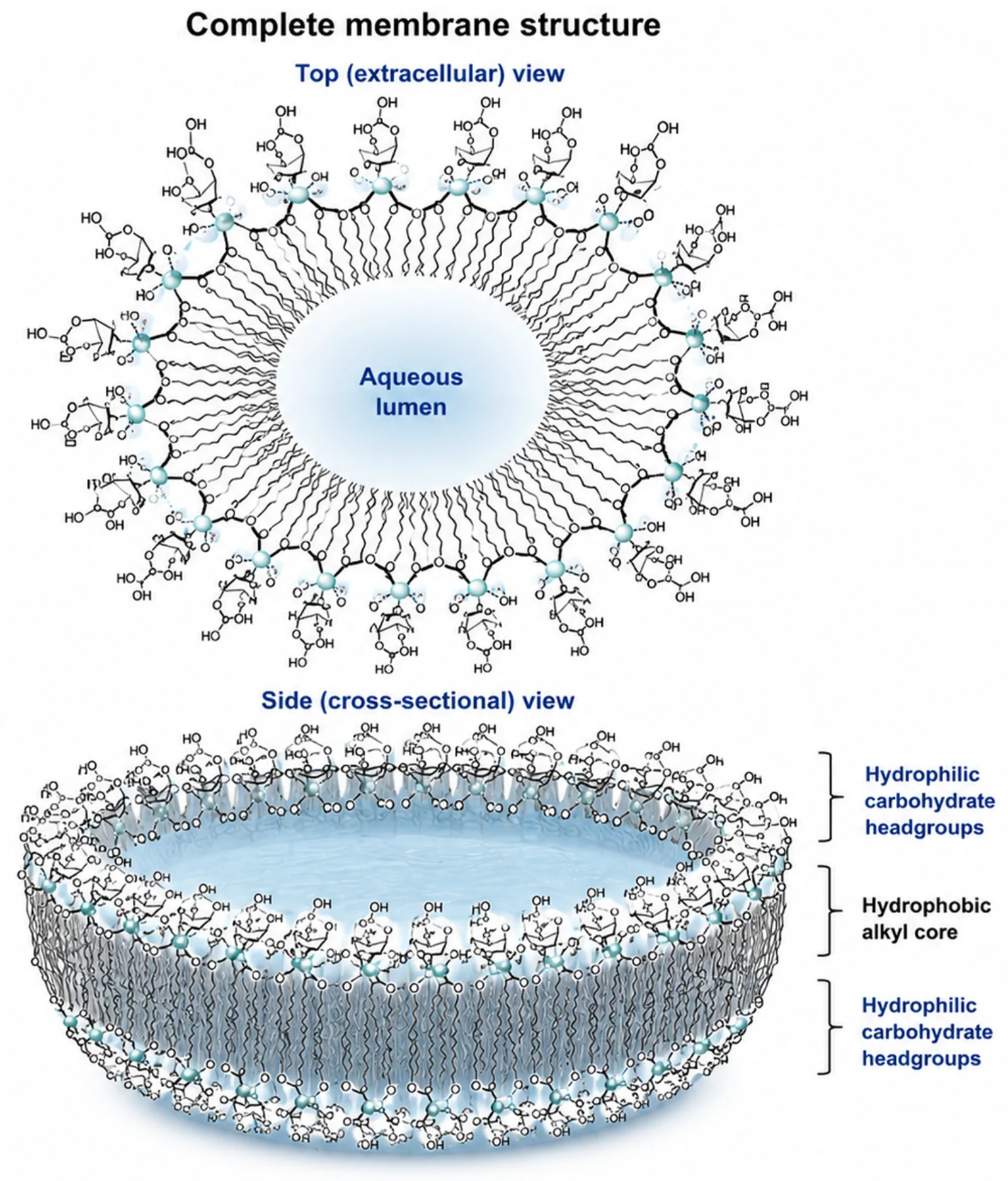
Proposed membrane architecture formed by borate-bridged amphiphile–carbohydrate conjugates. The figure presents a conceptual model of a protocellular membrane assembled from borate-associated amphiphile–carbohydrate conjugates. The upper panel shows a top (extracellular) view of a closed vesicle in which hydrophilic carbohydrate headgroups are exposed to the surrounding aqueous environment, while the lower panel illustrates a cross-sectional view highlighting the organization of the membrane. Long hydrophobic alkyl chains form a densely packed membrane core, whereas carbohydrate headgroups containing vicinal diols remain hydrated at both membrane interfaces. Reversible borate coordination between neighboring carbohydrate moieties is proposed to generate dynamic supramolecular interactions that reinforce membrane organization without preventing structural flexibility. This model represents a hypothetical prebiotic protocell membrane in which borate chemistry contributes simultaneously to membrane stability, compartmentalization, and adaptive self-assembly.

Figure 2 summarizes this conceptual model by illustrating how borate-mediated amphiphile–carbohydrate conjugates could self-assemble into closed membrane-bound compartments under plausible prebiotic conditions. The proposed architecture integrates three principal components: (i) hydrophobic alkyl chains that establish the membrane permeability barrier, (ii) highly hydrated carbohydrate and polyol head groups that interact with the surrounding aqueous environment, and (iii) reversible borate coordination at the membrane interface. Unlike permanent covalent cross-links, borate–diol interactions are dynamic and environmentally responsive, allowing membrane assemblies to maintain both structural integrity and adaptive flexibility. Although this model remains hypothetical, it provides a chemically plausible mechanism by which boron chemistry could have coupled membrane stabilization with molecular organization during the proposed Sulfur–Boron Era, preceding the evolution of modern phospholipid bilayers.

### Hydrogels as Transitional Structures Between Polyols, Protolipids, and Proto-RNA

One of the central challenges in origin-of-life research is explaining how simple organic molecules became organized into increasingly complex protocellular systems. Although the spontaneous self-assembly of amphiphilic molecules provides a plausible mechanism for compartmentalization, the transition from freely dispersed organic compounds to stable membrane-bound structures remains incompletely understood. We propose that an important intermediate stage involved the formation of highly hydrated supramolecular networks and hydrogel-like materials that bridged molecular synthesis, membrane assembly, and the emergence of proto-informational chemistry.

Hydrogels are three-dimensional molecular networks capable of retaining large quantities of water while maintaining structural integrity through hydrogen bonding, supramolecular interactions, reversible covalent linkages, and physical cross-linking. In modern biological systems, hydrogels are ubiquitous, functioning as extracellular matrices, biofilms, mucus layers, and intracellular phase-separated compartments [134,135,136,137]. Although these biological systems evolved much later, they demonstrate the remarkable ability of hydrated molecular networks to organize, concentrate, and stabilize complex chemical environments. Similar physicochemical principles may also have operated under prebiotic conditions [138,139,140,141].

Low-molecular-weight polyols possess physicochemical properties particularly favorable for the formation of hydrated molecular networks. Ethylene glycol, glycerol, tetritols, sugar alcohols, and simple carbohydrates contain multiple hydroxyl groups capable of establishing extensive hydrogen-bonding interactions [6,14,17,88]. In concentrated aqueous environments, these compounds can generate highly hydrated supramolecular assemblies that differ substantially from ordinary solutions. Such networks provide localized regions of reduced molecular mobility, enhanced molecular retention, and increased opportunities for intermolecular association and chemical reaction.

Borate chemistry may have further promoted the formation and stabilization of these hydrated networks. Borate ions readily form reversible cyclic esters with *cis*-vicinal diol groups present in sugars and polyols. Because individual borate species can reversibly coordinate multiple hydroxyl-containing molecules, dynamic cross-linked networks could arise spontaneously under suitable alkaline conditions [130,131,132,133,134]. These borate–polyol assemblies exhibit several characteristics associated with modern supramolecular hydrogels, including reversibility, environmental responsiveness, self-reorganization, and self-healing behavior.

Within the conceptual framework proposed here, hydrogels represent a plausible intermediate stage linking molecular synthesis with membrane formation and, ultimately, the emergence of proto-informational molecular networks. Rather than functioning as static materials, these hydrated assemblies could have concentrated organic molecules, promoted repeated molecular interactions, and provided chemically protected microenvironments in which increasingly complex amphiphiles, carbohydrates, and other prebiotic compounds accumulated and interacted. In this way, hydrogel-like systems may have facilitated the gradual transition from dispersed molecular mixtures to organized protomembranes and, eventually, to compartmentalized protocellular structures. The proposed evolutionary sequence may therefore be summarized as:

**Abiotic synthesis of polyols and carbohydrates** **↓** **hydrated polyol-rich networks and borate-stabilized hydrogels** **↓** **amphiphilic self-assembly and proto-lipid formation** **↓** **protomembranes** **↓** **compartmentalized proto-informational molecular networks** **↓** **early protocells**

This sequence represents a conceptual and experimentally testable model rather than an established evolutionary pathway. Nevertheless, it integrates well-established principles of supramolecular chemistry, borate coordination, hydrogel formation, and membrane self-assembly into a unified framework for investigating the emergence of cellular organization on the pre-phosphate Earth.

In this model, hydrogel matrices function as molecular concentration devices. Unlike dilute aqueous solutions, hydrogel environments can accumulate sugars, amino acids, amphiphiles, sulfur-containing molecules, and metal ions within restricted volumes. Such concentration effects increase the probability of chemical reactions and facilitate the emergence of increasingly complex supramolecular assemblies.

Hydrogels may also provide a natural bridge between polyol chemistry and membrane formation. Amphiphilic molecules generated within hydrogel matrices are expected to self-organize into localized lipid-rich domains [142,143]. As amphiphile concentration increases, these domains may evolve into micelles, bilayer fragments, and eventually closed membrane compartments. Rather than forming directly from dilute solution, protomembranes may therefore have emerged from pre-existing hydrated molecular networks already enriched in organic building blocks [142,143,144,145,146].

The relationship between hydrogels and informational chemistry is equally significant. Hydrated polymeric matrices can retain sugars, nucleobase precursors, sulfur-containing compounds, and borate-stabilized carbohydrates while simultaneously reducing diffusion rates. Such conditions favor repeated molecular interactions and selective stabilization processes. In this respect, hydrogels may have served as primitive reaction chambers in which the earliest proto-informational systems evolved [142,144,146].

An additional advantage of hydrogel systems is their dynamic nature. Unlike crystalline minerals, hydrogels remain soft, hydrated, and responsive to environmental changes. Cycles of hydration and dehydration, temperature fluctuations, pH shifts, and variations in ionic strength continuously remodel their internal organization [140,142,143,144]. Such behavior provides opportunities for molecular selection and adaptive self-organization without requiring genetically encoded mechanisms.

**Figure 3:**
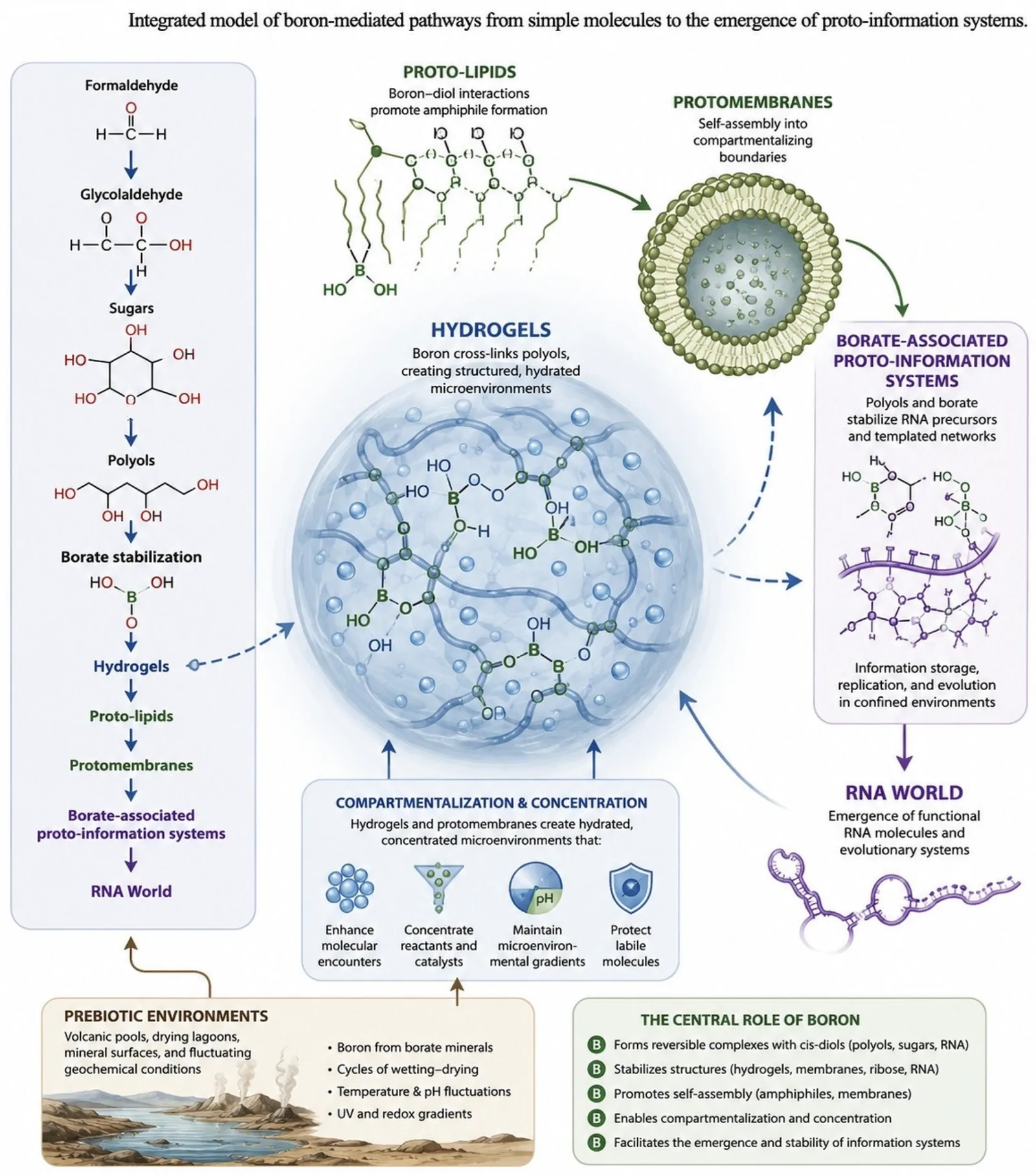
Integrated conceptual model of boron-mediated prebiotic evolution from simple organic molecules to proto-information systems. The figure illustrates a proposed evolutionary sequence in which boron chemistry provides a unifying framework linking abiotic organic synthesis with the emergence of protocellular organization and primitive information systems. Formaldehyde-derived carbohydrates and polyols produced through Butlerov-type reactions are stabilized by reversible borate complexation, facilitating the formation of proto-lipids, hydrogels, and self-assembled protomembranes. Hydrogels create hydrated and chemically structured microenvironments that promote molecular concentration, compartmentalization, and selective interactions, while borate-associated proto-information systems emerge through the stabilization and organization of ribose and other vicinal diol-containing molecules. Together, these interconnected processes culminate in the transition toward the RNA World. The lower panels summarize the geochemical settings that may have supported these processes and highlight the multiple physicochemical roles of boron in prebiotic evolution.

Figure 3 presents the central conceptual framework of this review by integrating the major stages of the proposed Sulfur–Boron Era into a single evolutionary pathway. Rather than acting solely as a stabilizer of ribose, boron is proposed to participate throughout prebiotic evolution by reversibly coordinating with polyols, carbohydrates, amphiphiles, and emerging informational molecules. This coordination chemistry promotes molecular stabilization, self-assembly, hydrogel formation, membrane organization, and the development of confined reaction environments where increasingly complex chemical processes could occur [142,143,144,145,146]. Within this model, hydrogels occupy a pivotal intermediate position by bridging dissolved organic molecules and membrane-bound compartments, thereby concentrating reactants and facilitating the emergence of primitive metabolic and informational networks. The figure emphasizes that the origin of protocellular systems was likely a gradual, interconnected process involving molecular self-organization rather than a series of isolated events. Although this evolutionary pathway remains hypothetical, it provides a coherent physicochemical framework linking boron-rich geochemical environments with the emergence of stable compartments, proto-information systems, and ultimately functional RNA-based biology.

Hydrogels therefore occupy a unique position within the proposed Sulfur–Boron Era model. They connect the molecular world of sugars and polyols with the supramolecular world of membranes and informational polymers. By concentrating reactants, stabilizing molecular assemblies, facilitating amphiphile organization, and promoting repeated chemical interactions, hydrogel-like systems may have provided the missing physicochemical bridge linking prebiotic organic chemistry to the emergence of protocellular evolution.

## 7. From Protomembranes to Proto-Informational Systems

The emergence of protomembranes established the physical basis for compartmentalization, but increasingly complex protocellular systems also required mechanisms capable of organizing, stabilizing, and transmitting chemical information [147,148]. We propose that membrane evolution and informational evolution were closely coupled processes that developed concurrently within dynamic prebiotic environments, rather than as independent evolutionary events.

Polyol-rich protomembranes could have generated localized microenvironments in which sugars, amphiphiles, amino acids, sulfur-containing compounds, and borate species accumulated at concentrations substantially higher than those in the surrounding aqueous medium [6,14,17]. Such compartmentalization would have increased the frequency of molecular interactions, promoted supramolecular self-assembly, and favored the emergence of increasingly organized chemical networks. These environments may therefore have provided the physicochemical setting for the development of proto-informational molecular networks that preceded phosphate-based genetic systems.

A major challenge for prebiotic chemistry is the limited bioavailability of soluble phosphate on the early Earth [66]. Although phosphorus was undoubtedly present, much of it was likely sequestered in poorly soluble mineral phases, restricting its participation in nucleotide synthesis and phosphodiester bond formation. Consequently, earlier stages of molecular evolution may have relied on alternative mechanisms for molecular organization before the appearance of phosphate-based informational polymers.

One plausible alternative involves borate-mediated molecular assemblies. Borate exhibits a strong affinity for cis-vicinal diol groups and readily forms reversible complexes with ribose and related carbohydrates [14,17,18,21,22,23]. Experimental studies have shown that borate stabilizes ribose in aqueous solution and influences the distribution of reaction products during carbohydrate synthesis [52,53,110]. We propose that these reversible borate–polyol interactions could also have generated dynamic molecular assemblies capable of selectively stabilizing particular carbohydrates while promoting transient supramolecular organization. Unlike modern RNA, such borate-associated networks would have remained highly dynamic, continuously assembling and dissociating in response to environmental conditions.

Sulfur-rich environments may have supported complementary forms of molecular organization. Volcanic and hydrothermal systems contained abundant sulfur species, including sulfide, polysulfides, thiosulfate, sulfite, and sulfate [149,150,151,152]. These compounds participate in diverse redox and coordination reactions that generate chemically rich reaction networks. Sulfur-containing amphiphiles, sulfated carbohydrates, sulfur-rich peptides, and mineral-associated sulfur compounds may therefore have contributed to chemically organized molecular assemblies through reversible covalent and non-covalent interactions [150,152].

Environmental pH was likely an important geochemical regulator of these processes. Alkaline conditions favored borate complexation and stabilization of ribose- and polyol-containing assemblies, whereas acidic sulfur-rich environments promoted sulfur-mediated reaction networks and sulfur-containing supramolecular organization [52,53,110]. Within the conceptual framework proposed here, these complementary chemical domains can be summarized as:

**Acidic environments** **↓** **Sulfur chemistry** **↓** **Chemically organized sulfur-associated molecular networks** **↓** **Alkaline environments** **↓** **Boron chemistry** **↓** **Borate-associated proto-informational molecular networks**

Although fundamentally different from modern nucleic acids, both types of molecular organization may have represented transitional stages between simple prebiotic chemistry and phosphate-based informational polymers. Their structural organization depended primarily on reversible chemical interactions rather than on permanent phosphodiester linkages.

The coexistence of proto-lipids, protomembranes, borate-stabilized carbohydrates, and sulfur-containing molecular networks suggests that compartment formation and informational organization evolved in parallel rather than sequentially [6,14,17,52,53,88,110]. Primitive membrane systems could have concentrated and protected reactive molecular components, while emerging proto-informational molecular networks enhanced chemical organization and increased the persistence of protocellular assemblies.

As phosphate gradually became more bioavailable, these dynamic molecular networks may have been progressively replaced by more stable nucleotide structures and phosphodiester-linked polymers. We therefore hypothesize that modern RNA emerged through a prolonged evolutionary transition from earlier sulfur- and boron-mediated systems of molecular organization operating within polyol-rich protomembrane compartments, rather than as an isolated chemical innovation [153,154,155].

Within this conceptual framework, the transition from protomembranes to proto-informational systems represents not a single evolutionary event but a gradual process of increasing compartmentalization, molecular concentration, supramolecular organization, and chemical stabilization. Together, these developments may have established the physicochemical foundation upon which phosphate-based informational polymers, primitive metabolic networks, and, ultimately, the first cellular life evolved.

## 8. Sulfur-Containing Molecules as Pre-Genetic Information Networks

One of the central principles of modern biology is that hereditary information is encoded in nucleic acids. Before the emergence of phosphate-based RNA and DNA, however, earlier forms of molecular organization may have performed some of the functions later associated with genetic systems [156,157,158,159]. We propose that, during the sulfur-rich pre-phosphate stage of Earth history, sulfur-containing molecules contributed to primitive mechanisms of molecular recognition, chemical communication, and compositional organization. Within this framework, sulfur chemistry is viewed not merely as a source of metabolic precursors but also as a potential contributor to the earliest stages of proto-informational molecular networks.

The concept of molecular information extends beyond nucleotide sequences. Information may also be expressed through molecular composition, stereochemistry, supramolecular organization, reaction pathways, and self-assembly behavior. Modern biological systems provide numerous examples in which biologically meaningful information is encoded outside nucleic acids. Glycans, membrane lipids, protein conformations, and redox signaling networks all participate in molecular recognition and cellular organization. Although these systems are products of biological evolution, they illustrate general physicochemical principles that may also have operated during prebiotic evolution.

Sulfolipids are particularly relevant in this context. These amphiphilic molecules contain sulfur-bearing head groups attached to hydrophobic chains and readily assemble into organized membrane structures [126,127,128]. Variations in chain length, degree of unsaturation, sulfur substitution, and head-group composition generate diverse membrane architectures that influence permeability, stability, phase behavior, and intermolecular interactions. We therefore suggest that analogous sulfur-containing amphiphiles could have generated compositionally distinct membrane assemblies on the prebiotic Earth. Such assemblies may have exhibited simple forms of compositional persistence during growth, fusion, and division, providing an elementary mechanism for maintaining structural organization without requiring sequence-based genetic polymers [160,161,162,163,164].

Sulfated carbohydrates and sulfur-containing polyols provide another potential level of molecular organization. In modern organisms, sulfation patterns within carbohydrates function as highly selective recognition elements, illustrating that biologically relevant information can be encoded through molecular architecture rather than nucleotide sequence alone [165,166,167,168]. Although these modern systems should not be regarded as direct models of prebiotic chemistry, they demonstrate the remarkable organizational capacity of sulfur-modified carbohydrates. We propose that structurally simpler sulfur-containing sugars and polyols may likewise have generated diverse supramolecular assemblies capable of selective molecular interactions and self-organization.

Sulfur-containing amino acids, thioesters, and sulfur-rich peptides may have contributed additional organizational complexity. Sulfur readily participates in redox chemistry, metal coordination, and reversible covalent reactions, enabling the formation of dynamic catalytic and regulatory networks [169,170]. Thioesters are particularly significant because they are energy-rich intermediates that have long been proposed as important components of prebiotic metabolism, potentially fulfilling energetic roles before the widespread utilization of phosphate chemistry [169,170,171,172,173]. Together, these sulfur-containing compounds may have coupled molecular organization with emerging energy-transduction processes.

A defining characteristic of sulfur-based molecular networks is their dynamic nature. Unlike modern nucleic acids, which preserve genetic information through stable covalent polymers, sulfur-containing systems would have relied primarily on reversible molecular interactions and continuously evolving chemical networks [174,175,176,177,178]. Self-assembled membranes, sulfur-rich hydrogels, sulfated carbohydrates, thioesters, and peptide assemblies may collectively have generated emergent organizational properties that persisted despite continual molecular turnover. In such systems, information would not have been localized within a single informational polymer but distributed throughout interacting molecular networks.

**Figure 4:**
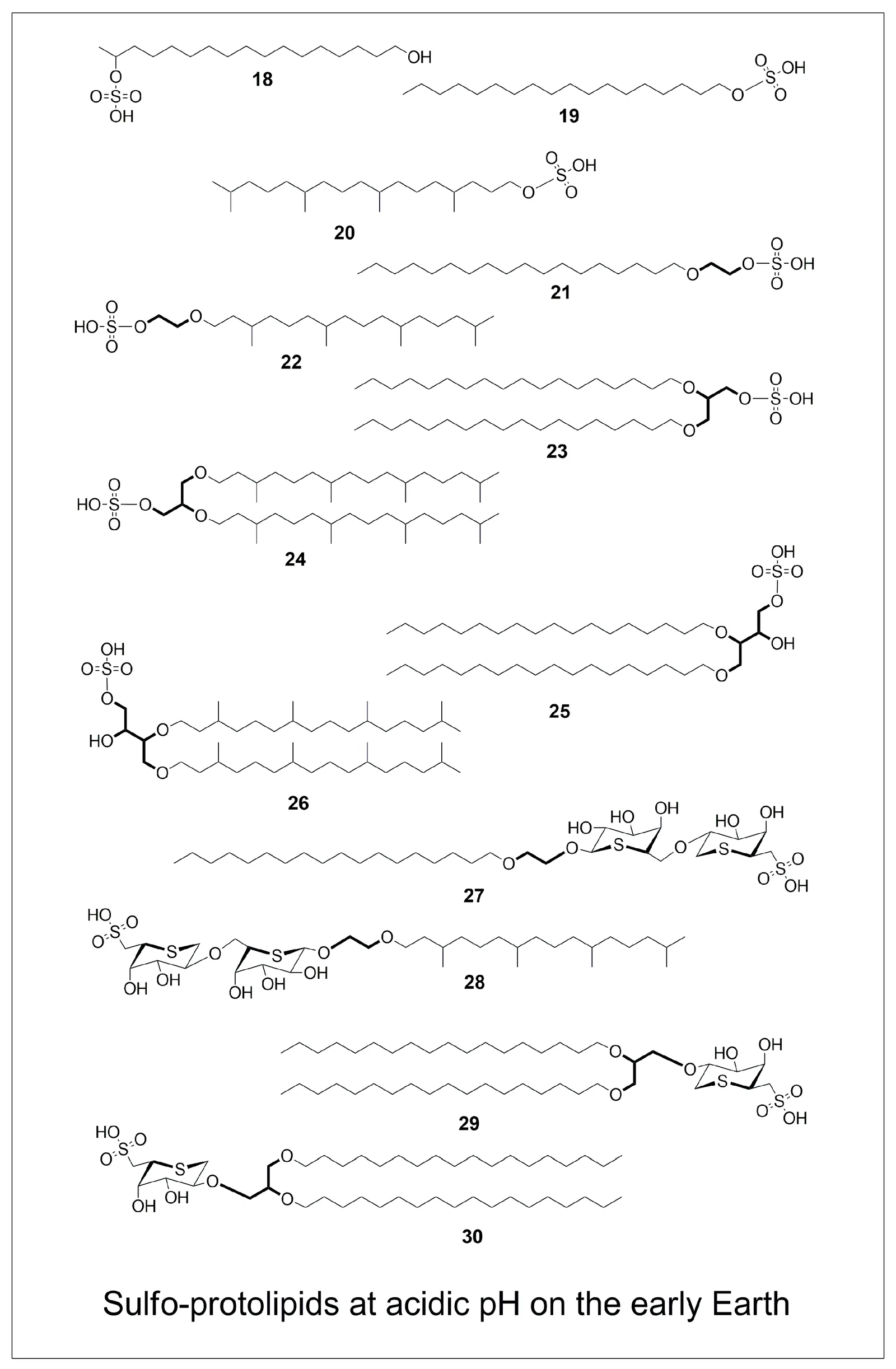
Representative sulfo-protolipids proposed for membrane formation under acidic prebiotic conditions. The figure illustrates a series of hypothetical sulfo-protolipids representing membrane-forming amphiphiles that may have been favored in acidic environments on the early Earth. The proposed structures include sulfate esters of long-chain alcohols, ether lipids, glycerol derivatives, archaeal-like isoprenoid ethers, and sulfur-containing carbohydrate amphiphiles. Sulfate groups provide strongly hydrophilic headgroups, whereas long hydrocarbon or isoprenoid chains form hydrophobic membrane domains capable of self-assembly into micelles, bilayers, or vesicle-like structures. Together, these molecules illustrate the structural diversity of sulfur-containing amphiphiles that could have contributed to primitive membrane organization before the widespread availability of phosphate-based lipids.

Figure 4 summarizes the proposed diversity of sulfur-containing amphiphiles (**18**–**30**) that may have been favored in acidic prebiotic environments. In contrast to the borate-associated amphiphiles proposed for alkaline settings, these hypothetical sulfo-protolipids are based on sulfate ester chemistry, which is expected to be more compatible with acidic hydrothermal conditions. The proposed structures combine hydrophobic alkyl or isoprenoid chains with sulfate-containing polar head groups, producing amphiphiles capable of spontaneous self-assembly into membrane-like structures (**18**–**30**). Some of these molecules resemble simplified structural analogues of modern archaeal ether lipids and glycolipids, illustrating the physicochemical feasibility of sulfur-containing membrane architectures rather than implying direct evolutionary continuity. The incorporation of carbohydrate-derived head groups further suggests that sulfur chemistry could have linked membrane assembly with the expanding diversity of prebiotic sugars and polyols. Although these structures remain hypothetical, they provide a conceptual framework for exploring how sulfur-rich geochemical environments may have promoted the emergence of adaptive protomembranes. Together with the borate-associated systems discussed in later sections, these proposed sulfo-protolipids support the broader hypothesis that distinct pH-dependent geochemical regimes on the early Earth generated complementary pathways for membrane evolution before the emergence of phosphate-dominated biology.

The coexistence of sulfur-rich molecular networks with borate-stabilized carbohydrates suggests an intriguing model for prebiotic molecular evolution. We propose that sulfur-containing systems promoted molecular recognition, compositional organization, and supramolecular assembly, whereas borate chemistry selectively stabilized particular carbohydrates, especially ribose and other cis-vicinal diol-containing polyols [1,6,17,88]. Acting together, these complementary processes could have reduced the effective chemical complexity of prebiotic mixtures while simultaneously increasing molecular organization and persistence. Within this conceptual framework, sulfur and boron fulfilled distinct but cooperative roles during early chemical evolution.

As borate-mediated molecular selection increasingly favored specific carbohydrate structures, particularly ribose and related polyols [6,18,19,20,21], progressively more stable supramolecular assemblies may have emerged. We therefore hypothesize that this transition provided a physicochemical bridge between dynamic sulfur-associated molecular networks and more organized borate-associated proto-informational molecular networks. As phosphate gradually became more bioavailable, these reversible systems may have been progressively replaced by chemically more stable nucleotide structures and phosphodiester-linked polymers, ultimately giving rise to the RNA-based informational systems characteristic of modern biology [179,180].

**Figure 5:**
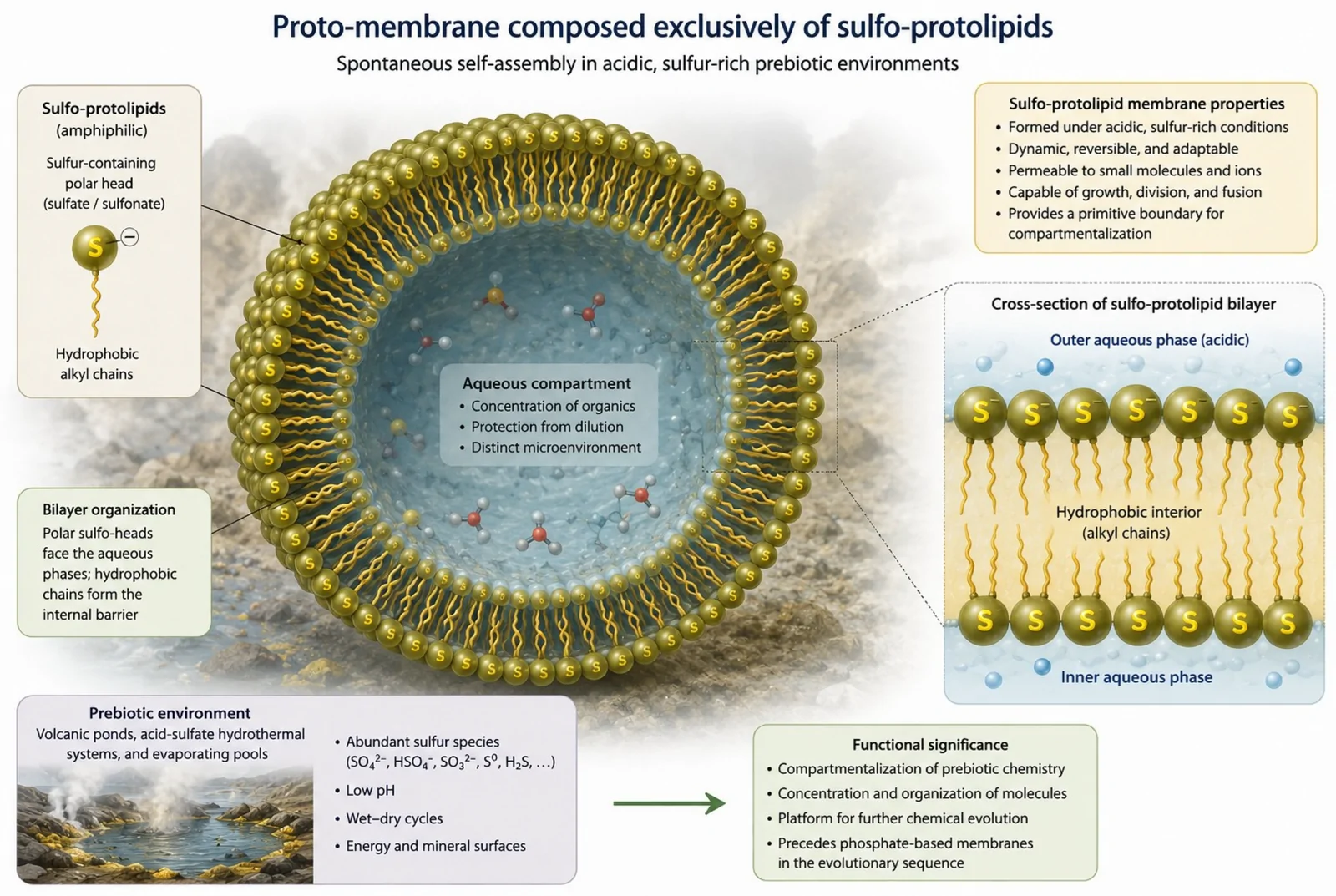
Conceptual model of a proto-membrane composed exclusively of sulfo-protolipids under acidic prebiotic conditions. The figure illustrates a hypothetical protocellular membrane assembled entirely from sulfur-containing amphiphiles in acidic, sulfur-rich environments on the early Earth. Sulfo-protolipids possessing hydrophilic sulfate or sulfonate headgroups and hydrophobic alkyl chains spontaneously organize into a bilayer surrounding an aqueous internal compartment. The cross-sectional view highlights the arrangement of polar sulfur-containing headgroups at the membrane–water interfaces and densely packed hydrophobic chains within the membrane core. The surrounding panels summarize the physicochemical properties of the membrane, the geochemical conditions favoring its formation, and its potential biological significance as an early compartment capable of concentrating organic molecules and supporting primitive chemical evolution.

From this perspective, the emergence of the RNA World was not the abrupt appearance of an entirely new informational system but rather the culmination of a prolonged evolutionary process involving increasing molecular organization, compartmentalization, and chemical stabilization. Sulfur-containing molecular networks may therefore represent an important organizational stage between non-living chemical self-organization and the evolution of true sequence-based genetic systems (Figure 5).

Within the Sulfur–Boron Era model proposed in this review, sulfur-containing molecular networks and borate-mediated molecular selection acted cooperatively to organize prebiotic chemistry before the widespread utilization of phosphate. Although this evolutionary scenario remains hypothetical, it integrates established principles of sulfur chemistry, borate coordination, supramolecular self-assembly, and membrane organization into a coherent and experimentally testable framework. Together, these complementary chemical systems may have established the physicochemical conditions from which phosphate-based informational polymers and, ultimately, the first cellular life emerged.

### Integrated Evolutionary Scenario

The evolutionary sequence proposed in this review can be summarized as follows:

**Sulfur Earth** **↓** **Sulfur-containing lipids** **↓** **Sulfur-containing sugars and polyols** **↓** **Sulfur-containing amino acids** **↓** **Sulfopeptides and thioester networks** **↓** **Sulfur-rich organizational networks** **↓** **Borate selection and stabilization** **↓** **Polyol-rich proto-lipids** **↓** **Proto-membranes** **↓** **Borate-associated proto-informational systems** **↓** **RNA World** **↓** **Phosphate-based biology**

In this scenario, phosphorus is not viewed as the starting point of molecular evolution but rather as the culmination of a prolonged period of chemical innovation involving sulfur-rich reaction networks, borate-mediated molecular selection, compartmentalization, and progressive increases in molecular stability. The emergence of phosphate chemistry therefore represents the final stage of a continuous evolutionary transition from dynamic prebiotic molecular organization to the genetically encoded biology observed today.

The model does not imply that sulfur- or boron-based systems were direct ancestors of RNA or modern metabolic pathways. Rather, it proposes that these complementary chemistries provided successive physicochemical frameworks that progressively increased molecular organization, stability, and functional complexity before the widespread incorporation of phosphate into biological systems. Because each stage of the proposed sequence is grounded in experimentally accessible chemical processes—including sulfur redox chemistry, borate coordination, amphiphile self-assembly, and supramolecular organization—the overall framework generates testable hypotheses for future laboratory investigations of prebiotic evolution.

Table 3 is retained in this section because it provides a concise comparison of plausible sulfur-era analogues for several biological functions that later became dominated by phosphate chemistry. Rather than presenting sulfur chemistry as an alternative to phosphate-based biology, the table emphasizes the *Sulfur–Boron Era* as a transitional evolutionary framework linking early prebiotic chemical organization with the emergence of modern phosphate-dependent cellular systems.

Figure 5 presents a conceptual model for one of the earliest membrane architectures that could have emerged in acidic sulfur-rich environments before the widespread incorporation of phosphate-containing lipids. Sulfo-protolipids are proposed to self-assemble spontaneously through hydrophobic interactions, producing closed vesicular compartments with aqueous interiors capable of concentrating dissolved organic molecules. Sulfate and sulfonate headgroups remain exposed to the surrounding aqueous phase, while the hydrocarbon chains form a continuous permeability barrier that separates the internal microenvironment from the external solution. Such membranes would have been dynamic and reversible, allowing growth, fusion, division, and continual structural reorganization in response to environmental fluctuations. Within the proposed Sulfur–Boron Era model, these sulfur-based membranes represent an evolutionary pathway complementary to borate-associated membrane systems that developed under alkaline conditions. Together, these two pH-dependent membrane strategies illustrate how distinct geochemical environments may have generated different classes of primitive amphiphilic assemblies before the eventual emergence of phosphate-based biological membranes. Although hypothetical, this model provides a plausible physicochemical framework linking sulfur-rich hydrothermal settings with the earliest stages of protocellular compartmentalization and prebiotic chemical evolution.

## 9. Neutral pH Domain: Polyol–Fatty Acid Proto-Lipids and Transitional Membranes

Between the acidic sulfur-dominated environments and the alkaline borate-rich systems proposed for the pre-phosphate Earth, a broad range of near-neutral aqueous environments (approximately pH 6.5–7.5) likely provided chemically favorable settings for the formation of mixed amphiphilic systems [181,182,183,184]. Shallow ponds, coastal lagoons, tidal flats, and many freshwater hydrothermal environments probably maintained near-neutral conditions for extended periods, allowing diverse classes of organic molecules to coexist and interact.

Unlike acidic environments, where sulfur-containing amphiphiles may have been favored, or alkaline systems, where borate complexation strongly influenced molecular selection, near-neutral conditions were well suited for interactions between fatty acids and low-molecular-weight polyols. Fatty acids generated through abiotic synthesis or delivered by extraterrestrial sources could have reacted with ethylene glycol, glycerol, tetritols, and related polyols to produce ester- and ether-linked amphiphiles with greater structural diversity than free fatty acids alone [1,2,3,7,9].

We propose that these mixed polyol–fatty acid proto-lipids possessed important physicochemical advantages over membranes composed exclusively of fatty acids. Incorporation of polyol backbones would have increased opportunities for hydrogen bonding, improved hydrophilic–hydrophobic coupling, enhanced packing of hydrocarbon chains, and potentially reduced membrane permeability while increasing mechanical stability [6,14,17]. As a result, mixed amphiphilic membranes may have displayed greater resistance to fluctuations in ionic strength, hydration, temperature, and other environmental variables than simple fatty acid vesicles.

Near-neutral pH also favors the spontaneous self-assembly of amphiphilic mixtures into bilayers, vesicles, and related supramolecular structures. Under these conditions, borate ester formation is less extensive than in alkaline environments, while sulfur chemistry is less dominant than under acidic conditions. Consequently, membrane organization would have depended primarily on amphiphilic self-assembly, hydrogen bonding, hydration, and hydrophobic interactions [163,185,186,187]. Experimental studies have shown that chemically mixed amphiphilic systems frequently exhibit broader stability ranges and greater robustness than membranes composed of a single amphiphile class [1,2,3,4,7,9]. We therefore suggest that near-neutral environments were particularly favorable for the progressive evolution of increasingly stable and compositionally diverse protomembranes.

Within the conceptual framework developed in this review, the neutral pH domain represents a physicochemical bridge linking sulfur-rich and borate-rich geochemical environments. Sulfur-containing amphiphiles generated under acidic conditions and borate-stabilized polyols originating in alkaline settings could have converged within near-neutral environments, where mixed amphiphilic systems underwent further self-assembly, molecular selection, and structural diversification [1,2,6,14,17,188,189]. Rather than representing an independent evolutionary pathway, the neutral domain served as the principal setting in which complementary products of sulfur and boron chemistry could interact and evolve toward increasingly complex proto-lipids and protomembranes. The relationships among the three proposed pH-dependent geochemical domains are summarized in Table 4.

We propose that the neutral pH domain served as the principal evolutionary bridge between the chemically specialized membrane systems of acidic sulfur-rich environments and alkaline borate-rich settings. Rather than representing an independent evolutionary pathway, mixed polyol–fatty acid membranes may have integrated molecular components originating from both geochemical domains, thereby facilitating the transition toward increasingly stable and compositionally diverse protocellular membranes [188,189,190,191]. Within this framework, near-neutral environments provided optimal conditions for the convergence of sulfur chemistry, polyol-based amphiphiles, and borate-stabilized molecular building blocks into progressively more complex membrane systems.

**Figure 6:**
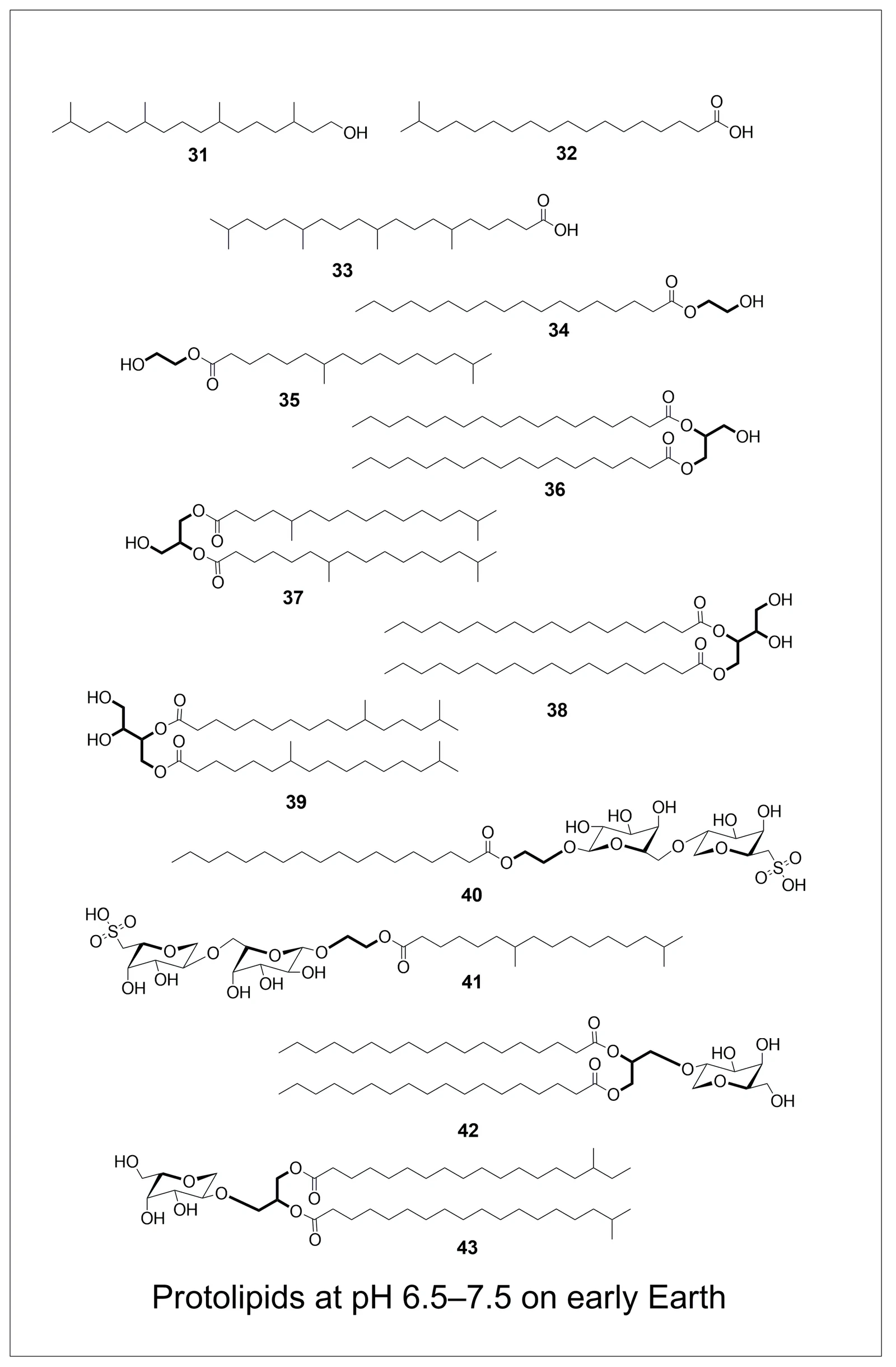
Representative protolipids proposed for self-assembly under near-neutral prebiotic conditions (pH 6.5–7.5). The figure presents a series of hypothetical protolipids that may have been favored under mildly acidic to neutral environments on the early Earth. The proposed molecules include long-chain alcohols, fatty acids, mono- and diacylglycerols, glycerol esters, glycolipids, and mixed carbohydrate-containing amphiphiles. Compared with sulfur-rich amphiphiles favored under acidic conditions and borate-associated systems expected in alkaline environments, these compounds represent an intermediate class of membrane-forming molecules stabilized primarily through hydrophobic interactions and hydrogen bonding. Their structural diversity illustrates several plausible pathways toward increasingly complex amphiphilic assemblies capable of forming primitive bilayers and vesicular compartments.

Figure 6 illustrates this proposed transitional stage in the pH-dependent evolution of prebiotic membranes. Under near-neutral conditions, neither sulfate- nor borate-mediated chemistry is expected to dominate completely, permitting a chemically heterogeneous population of amphiphiles (**31**–**43**) to coexist and participate in membrane assembly. Simple fatty acids and fatty alcohols likely constituted the earliest membrane-forming components, whereas subsequent esterification with glycerol and the incorporation of carbohydrate-derived head groups generated amphiphiles with increased structural diversity and improved membrane-forming properties. Several of the proposed molecules resemble simplified structural analogues of modern glycerolipids and glycolipids, illustrating plausible physicochemical intermediates rather than direct evolutionary precursors.

Because near-neutral environments favor extensive hydrogen bonding, hydration, and amphiphilic self-assembly, these proto-lipids could have organized into dynamic bilayers capable of growth, fusion, molecular exchange, and spontaneous reorganization while remaining sufficiently stable to maintain compartmentalization. Although this model remains hypothetical, it provides a chemically plausible mechanism by which changing geochemical conditions could have driven the progressive evolution of membrane architecture. Within the Sulfur–Boron Era framework proposed here, the neutral pH domain represents the critical transitional stage linking sulfur-dominated membrane systems in acidic environments with borate-associated amphiphiles in alkaline settings, ultimately leading to the emergence of increasingly sophisticated protocellular membranes before the evolution of phosphate-based phospholipids.

The three environmental domains summarized in Table 5 represent complementary physicochemical settings that may have coexisted on the prebiotic Earth rather than successive, mutually exclusive stages of evolution. We propose that each pH domain favored the formation of distinct classes of amphiphilic molecules and membrane architectures through differences in molecular stability, reaction pathways, and supramolecular self-assembly. Acidic environments promoted sulfur-rich chemistry and the formation of sulfur-containing proto-lipids, whereas alkaline environments favored borate-mediated stabilization and molecular selection of sugars and polyols, facilitating the emergence of borate-associated amphiphiles and increasingly organized protomembranes. Between these two end-member systems, near-neutral environments provided optimal conditions for interactions between fatty acids and low-molecular-weight polyols, leading to mixed amphiphilic membranes with enhanced structural diversity, improved stability, and greater membrane-forming capacity.

Rather than representing isolated evolutionary pathways, these three geochemical domains likely functioned as interconnected components of a dynamic planetary chemical system. Continuous exchange of molecules among acidic, neutral, and alkaline environments would have promoted chemical diversification, molecular selection, and the gradual emergence of increasingly sophisticated membrane architectures. Within the Sulfur–Boron Era framework proposed here, the interplay among these complementary pH-dependent domains provides a plausible physicochemical basis for the evolution of proto-lipids and protomembranes before the eventual emergence of phosphate-based biological membranes.

Rather than representing independent evolutionary pathways, these membrane systems may have exchanged molecular components through dynamic geological processes, including wetting–drying cycles, hydrothermal circulation, volcanic activity, evaporation, and local fluctuations in pH and temperature. We propose that such continual physicochemical exchange promoted molecular selection, membrane remodeling, and progressive increases in structural complexity. Within this conceptual framework, pH emerges as a fundamental geochemical organizer of prebiotic membrane evolution, influencing not only molecular stability but also the distribution, composition, self-assembly, and adaptive properties of primitive amphiphilic systems.

Accordingly, the model proposed here suggests that the earliest protocellular membranes did not arise from a single class of amphiphiles but evolved through the convergence of sulfur-containing, polyol-based, and borate-associated membrane systems. The integration of these complementary chemistries provides a plausible evolutionary bridge between simple prebiotic amphiphiles and the increasingly sophisticated membrane architectures capable of supporting compartmentalization, molecular organization, and protocellular evolution before the emergence of phosphate-based phospholipids.

**Figure 7:**
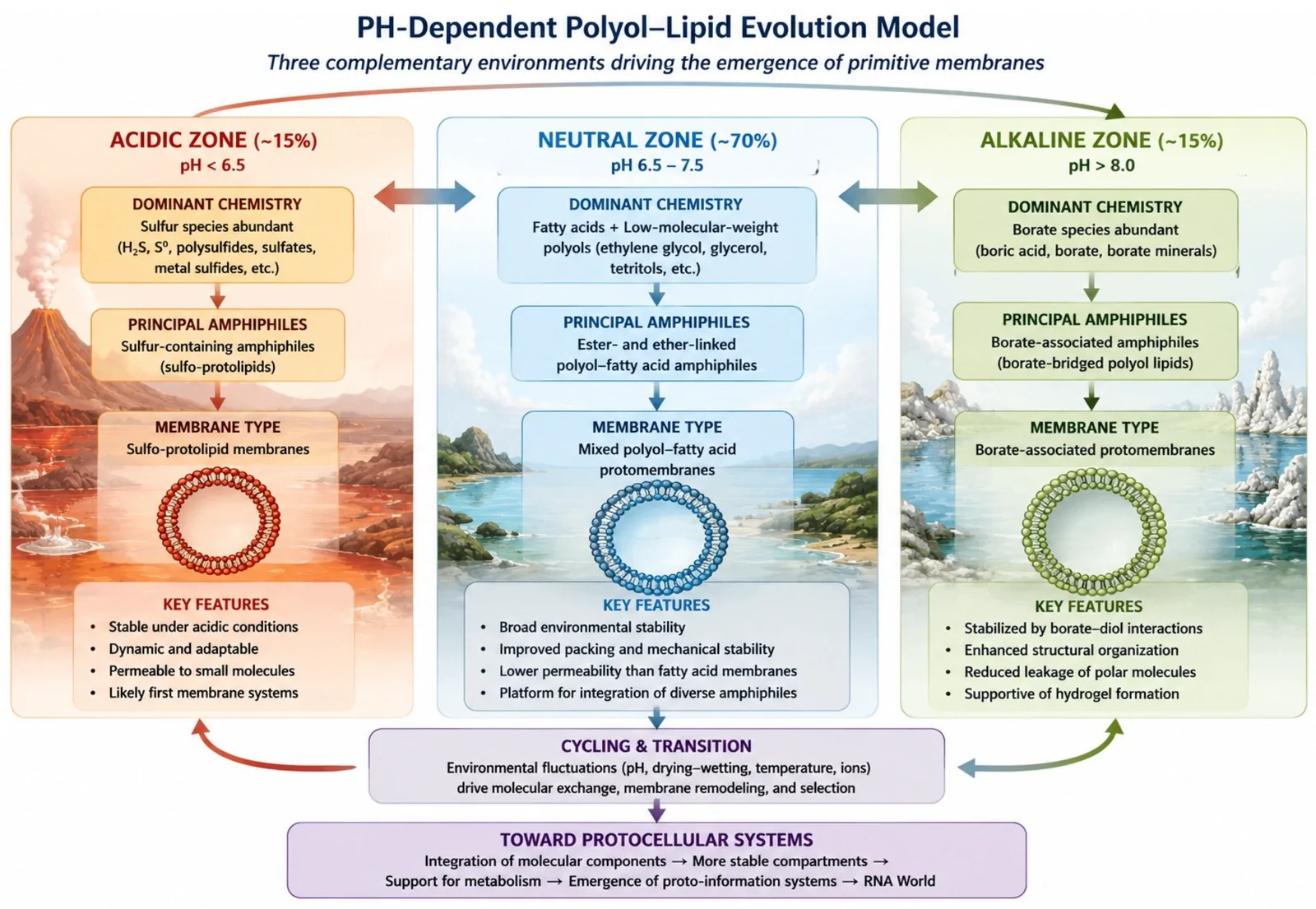
pH-dependent model for the evolution of polyol-based protolipids and primitive membranes on the early Earth. The figure presents a conceptual model illustrating how distinct pH regimes on the early Earth may have promoted different pathways of amphiphile formation and membrane evolution. Three complementary geochemical domains are proposed: an acidic sulfur-rich environment favoring sulfo-protolipids, a broad near-neutral environment supporting mixed polyol–fatty acid amphiphiles, and an alkaline boron-rich environment promoting borate-associated polyol lipids. Each chemical domain is characterized by its dominant molecular interactions, representative membrane-forming amphiphiles, and corresponding membrane architectures. Environmental cycling between these pH regimes is proposed to drive molecular exchange, membrane remodeling, and the progressive evolution of increasingly stable protocellular systems that ultimately supported the emergence of proto-information systems and the RNA World.

Figure 7 summarizes one of the central concepts developed in this review: primitive membrane evolution was likely driven not by a single universal pathway but by the interplay of multiple pH-dependent geochemical environments operating simultaneously on the early Earth. Acidic hydrothermal systems may have favored sulfur-containing amphiphiles and sulfur-mediated membrane organization, whereas near-neutral environments provided optimal conditions for the accumulation, modification, and self-assembly of fatty acids, glycerol derivatives, and low-molecular-weight polyols. In alkaline boron-rich settings, reversible borate–diol interactions introduced an additional level of molecular organization by selectively stabilizing polyols and carbohydrates while promoting the formation of borate-associated amphiphiles and increasingly organized protomembranes.

Rather than functioning as isolated evolutionary routes, these three geochemical domains were probably interconnected through planetary-scale geological cycling, including hydrothermal circulation, wetting–drying episodes, evaporation, mineral transport, and fluctuations in pH, temperature, and ionic composition. Such continual exchange would have promoted chemical diversification, molecular selection, and the gradual emergence of increasingly efficient membrane architectures. Although this integrated model remains hypothetical, it provides a coherent and experimentally testable framework linking sulfur chemistry, polyol-based amphiphiles, borate-mediated molecular selection, and supramolecular self-assembly. Within the proposed Sulfur–Boron Era, these complementary processes may have generated progressively more stable protocellular compartments capable of supporting proto-metabolic chemistry, proto-informational molecular networks, and, ultimately, the transition to the phosphate-dependent RNA World.

### 9.1. Borate-Bridged Calditol-Containing Tetraether Protolipids

Borate-bridged amphiphile–carbohydrate conjugates provide a conceptual link between simple prebiotic amphiphiles and the highly specialized membrane architectures found in modern Archaea [2,192,193,194,195,196]. Earlier sections considered reversible borate complexes formed between low-molecular-weight polyols and amphiphilic molecules, illustrating how borate ester formation could stabilize cis-vicinal diol-containing head groups while preserving the amphiphilic properties required for membrane self-assembly. We propose that these dynamic conjugates represent plausible intermediates in the evolutionary transition from simple molecular aggregates to increasingly robust membrane systems.

This concept can be extended to archaeal-type ether lipids containing calditol or related polyol head groups. Calditol-containing diether and tetraether lipids possess multiple cis-vicinal diol groups that provide favorable coordination sites for borate, whereas their ether-linked isoprenoid chains confer exceptional chemical, thermal, and hydrolytic stability [118,197,198]. Although these lipids are products of biological evolution, they serve as valuable structural analogues demonstrating how polyol-rich membrane architectures can support highly stable membrane systems. We therefore hypothesize that reversible borate coordination between neighboring calditol head groups could generate dynamic supramolecular interactions that reinforce membrane organization without forming permanent covalent cross-links.

Unlike conventional phospholipid membranes, these hypothetical borate-associated tetraether protomembranes combine several complementary stabilization mechanisms: hydrolysis-resistant ether linkages, membrane-spanning tetraether cores, tightly packed isoprenoid chains, extensive hydrogen bonding among polyol head groups, and reversible borate-mediated supramolecular organization. Together, these features could enhance membrane cohesion, reduce proton leakage and water permeability, and improve resistance to thermal and chemical stress while preserving the dynamic flexibility required for adaptive self-assembly.

**Figure 8:**
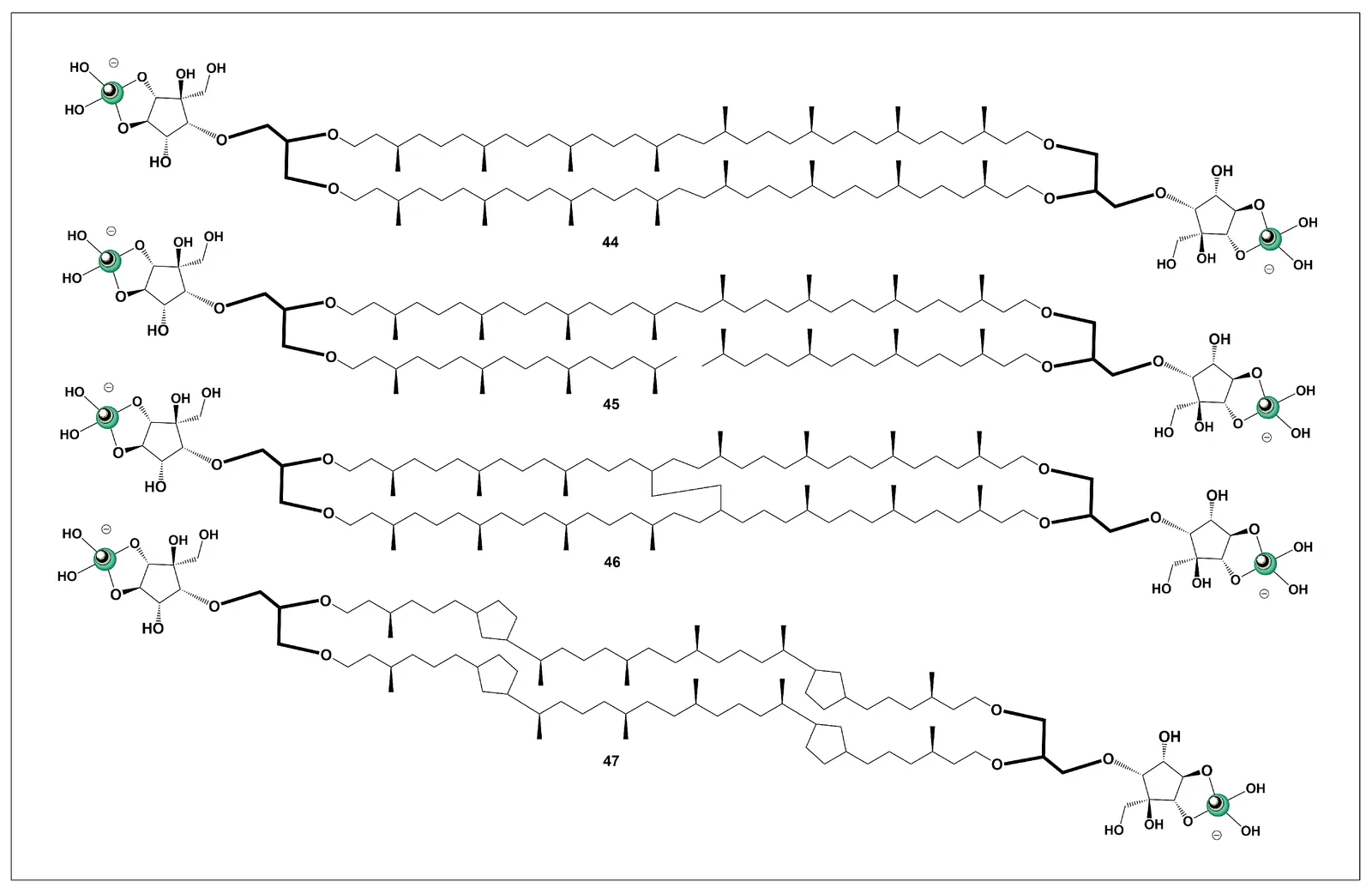
Proposed borate-bridged calditol-containing tetraether protolipids as models for archaeal-like prebiotic membranes. The figure illustrates a series of hypothetical borate-associated tetraether lipids based on archaeal membrane architecture. Each structure contains membrane-spanning ether-linked biphytanyl chains terminated by calditol headgroups capable of forming reversible cyclic borate esters through coordination with vicinal hydroxyl groups. The proposed molecules include simplified tetraether lipids as well as variants containing cyclopentane rings within the hydrophobic core, representing structural adaptations that enhance membrane rigidity and thermal stability in modern archaea. Together, these borate-bridged tetraether lipids provide conceptual models for highly stable protomembranes that integrate archaeal ether chemistry with reversible borate–polyol interactions.

Within the conceptual framework proposed here, the borate-bridged calditol-containing tetraether proto-lipids (**44**–**47**, Figure 8) represent a plausible physicochemical bridge between simple borate-associated amphiphiles and the highly specialized membranes of extant archaea. In addition to reinforcing membrane structure, reversible borate coordination may have promoted molecular clustering, selective compartmentalization, and local concentration of reactive organic molecules at membrane interfaces, thereby facilitating increasingly complex prebiotic reaction networks.

Although these proposed structures remain hypothetical, they considerably broaden the potential role of boron in prebiotic evolution. Beyond stabilizing carbohydrates such as ribose, borate may also have contributed directly to membrane organization by dynamically linking polyol-containing amphiphiles into more cohesive supramolecular assemblies. This model therefore extends the role of boron from molecular selection in solution to the stabilization and functional organization of primitive membrane systems.

Figure 8 illustrates this extension of the borate-mediated membrane hypothesis to archaeal-like membrane architectures. Unlike fatty acid membranes, archaeal tetraether lipids form membrane-spanning monolayers connected by chemically stable ether linkages that exhibit exceptional resistance to hydrolysis, elevated temperatures, and extreme pH. In the proposed model, calditol head groups provide multiple cis-vicinal diol sites capable of reversibly coordinating borate on both membrane surfaces (**44**–**47**). Because borate–diol interactions are dynamic, membrane reinforcement can occur without rigid covalent cross-linking, allowing simultaneous stabilization and structural adaptability. The cyclopentane-containing analogues further illustrate how increased membrane packing may have enhanced stability under hydrothermal conditions. Although this model is hypothetical, it provides a chemically plausible mechanism by which reversible borate coordination could have contributed to the evolution of exceptionally robust protocellular membranes before the emergence of modern phospholipid-based cellular membranes.

### 9.2. Amphiphile–Carbohydrate Conjugates and Protocell Relevance

Borate-bridged amphiphile–carbohydrate conjugates (**48** and **49**) provide a conceptual model illustrating how reversible borate chemistry may have linked simple carbohydrates with primitive membrane-forming amphiphiles on the prebiotic Earth [6,14,17,88]. Through reversible ester formation with cis-vicinal diol groups, borate can associate hydrophilic polyols or carbohydrates with hydrophobic amphiphilic molecules, generating dynamic hybrid structures capable of self-assembly while retaining the environmental responsiveness characteristic of borate coordination.

These conjugates combine hydrophobic membrane-forming domains with highly hydrated carbohydrate head groups, producing amphiphilic architectures that may have enhanced membrane cohesion, surface hydration, selective permeability, and supramolecular organization under fluctuating prebiotic conditions [88]. Because borate complexation is reversible and strongly dependent on pH, ionic composition, and borate availability, these assemblies would have remained dynamic rather than permanently cross-linked, allowing continual structural adaptation in response to changing geochemical environments.

Particularly noteworthy are ribose-containing conjugates, which establish a conceptual connection between membrane assembly and the carbohydrate that later became central to RNA chemistry. Experimental studies have demonstrated that borate stabilizes ribose under plausible prebiotic conditions [14,17,18,21,22,23,52,53]. We therefore propose that reversible borate-mediated association of ribose with amphiphilic molecules could have provided a physicochemical link between compartment formation and the progressive organization of carbohydrate-rich molecular systems. At the same time, conjugates containing polyols such as mannitol illustrate that this chemistry is not restricted to ribose but may represent a more general mechanism for organizing diverse polyol- and carbohydrate-containing amphiphiles into membrane-associated supramolecular assemblies.

Although these proposed structures remain hypothetical, they considerably broaden the potential role of boron in prebiotic evolution. Beyond stabilizing carbohydrates in solution, borate-mediated amphiphile–carbohydrate conjugation may have promoted molecular organization at membrane interfaces, facilitated compartment formation, and enhanced the structural complexity of primitive protocellular systems. Within the conceptual framework proposed here, these conjugates represent a plausible intermediate stage linking borate-mediated molecular selection with the emergence of increasingly organized protomembranes and proto-informational molecular networks.

**Figure 9:**
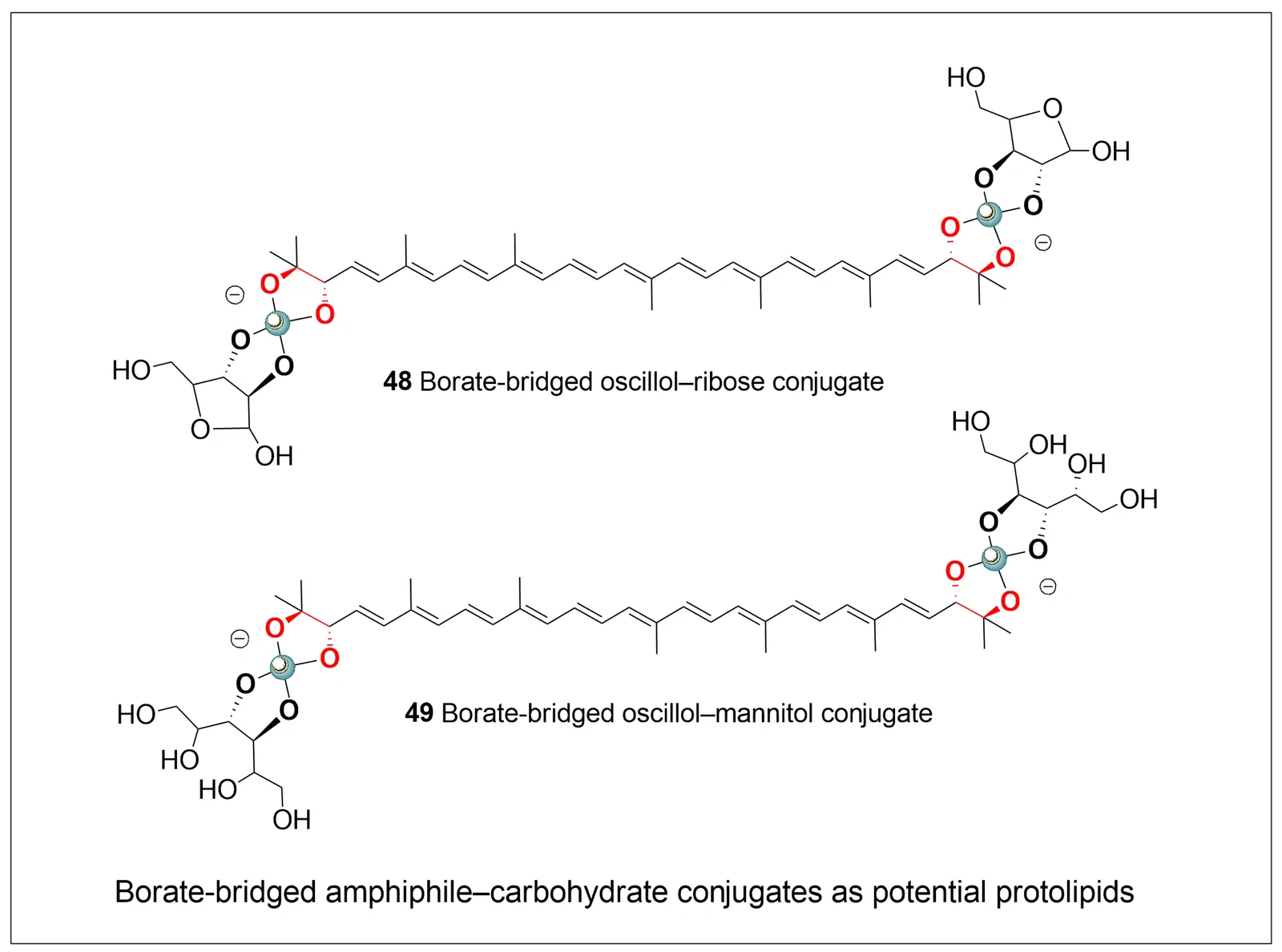
Proposed borate-bridged amphiphile–carbohydrate conjugates as potential protolipids. The figure illustrates two hypothetical borate-mediated amphiphile–carbohydrate conjugates representing possible intermediates in prebiotic membrane evolution. Compound 48 depicts an oscillol–ribose conjugate, while compound 49 shows an analogous oscillol–mannitol conjugate. In both structures, reversible cyclic borate esters are formed through coordination of borate with vicinal diol groups present in the carbohydrate moieties and terminal hydroxyl groups of the amphiphilic xanthophyll oscillol. These hybrid molecules combine an extended hydrophobic polyene chain with highly hydrophilic carbohydrate headgroups, producing amphiphilic architectures capable of molecular self-assembly. The structures are presented as conceptual models illustrating how borate chemistry could have coupled carbohydrate stabilization with membrane formation during the proposed pre-phosphate stage of chemical evolution.

Figure 9 illustrates one possible mechanism by which reversible borate coordination could integrate two essential components of prebiotic chemistry: membrane-forming amphiphiles and polyol-rich carbohydrates [88]. Oscillol provides an extended hydrophobic scaffold capable of participating in membrane assembly, whereas ribose and mannitol contribute highly hydroxylated head groups containing multiple cis-vicinal diol groups that readily undergo reversible borate complexation. The resulting amphiphile–carbohydrate conjugates remain sufficiently dynamic to undergo continual molecular exchange while simultaneously increasing membrane organization and interfacial stability. Although this model is hypothetical, it illustrates how borate chemistry could have coupled membrane assembly with carbohydrate stabilization, thereby providing a plausible physicochemical bridge between compartment formation and the emergence of increasingly organized prebiotic molecular systems.

This combination generates amphiphilic conjugates with pronounced structural polarity and the potential to self-assemble into membrane-associated supramolecular architectures. The ribose-containing conjugate is particularly noteworthy because it establishes a conceptual connection between membrane formation and the carbohydrate that later became central to RNA chemistry. Experimental studies have shown that borate stabilizes ribose under plausible prebiotic conditions [14,17,18,21,22,23,52,53]. We therefore propose that reversible borate-mediated association of ribose with amphiphilic molecules may have simultaneously promoted membrane organization and stabilized ribose-containing molecular building blocks within the same prebiotic microenvironment. In contrast, the mannitol-containing conjugate illustrates that this chemistry is not unique to ribose but may represent a more general mechanism for organizing a wide range of polyols into membrane-associated amphiphilic systems [199,200,201,202,203,204]. Although these conjugates remain hypothetical, they support the broader hypothesis that reversible borate coordination could have coupled carbohydrate stabilization, amphiphile self-assembly, and molecular compartmentalization within a common physicochemical framework, thereby facilitating the emergence of increasingly organized protocellular systems during the proposed Sulfur–Boron Era.

**Figure 10:**
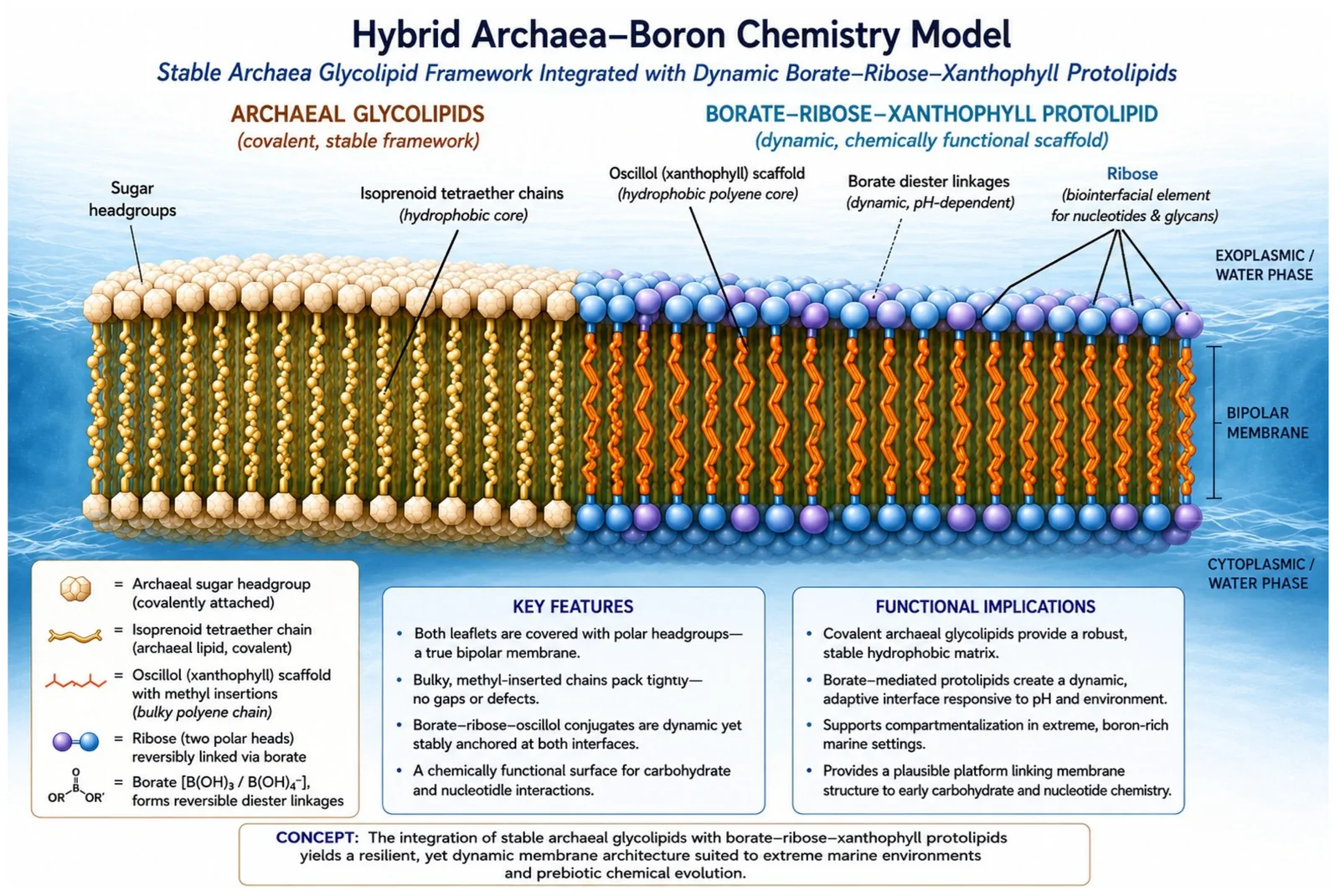
Hybrid Archaea–Boron Chemistry Model. Conceptual comparison between the covalently stabilized glycolipid membrane of Archaea and a hypothetical borate–ribose–xanthophyll protolipid membrane proposed for prebiotic evolution. Archaeal glycolipids form a rigid tetraether membrane stabilized by covalent linkages, whereas the proposed protolipid membrane consists of oscillol-derived hydrophobic chains bearing ribose polar headgroups on both membrane surfaces. Reversible borate diester bridges connect adjacent ribose residues, producing dynamic amphiphilic conjugates with two hydrophilic termini while preserving a tightly packed, defect-free hydrophobic core. The model illustrates how borate-mediated supramolecular organization could generate chemically adaptive yet structurally stable protocellular membranes capable of supporting compartmentalization and early carbohydrate- and nucleotide-related chemistry before the emergence of modern phospholipid membranes.

Figure 10 presents a conceptual comparison between two membrane architectures: a modern archaeal glycolipid membrane (left) and a hypothetical borate–ribose–xanthophyll proto-lipid membrane (right). The archaeal membrane is depicted as a covalently assembled lipid framework composed of membrane-spanning tetraether isoprenoid chains and carbohydrate head groups, illustrating the exceptional structural stability of archaeal membranes under extreme environmental conditions. This membrane serves as a structural analogue rather than a direct evolutionary model for prebiotic systems.

In the proposed proto-lipid membrane, oscillol (xanthophyll)-derived hydrophobic scaffolds bearing methyl-branched hydrocarbon chains form a densely packed hydrophobic core with minimal structural voids [88]. At both membrane surfaces, ribose molecules function as highly hydrated polar head groups and are reversibly interconnected through borate diester linkages, generating amphiphilic conjugates with two hydrophilic termini. Because borate–diol interactions remain reversible, the membrane interface would be capable of continual molecular reorganization while preserving overall membrane cohesion. Such dynamic interfaces could facilitate interactions with carbohydrates, nucleoside precursors, and other cis-vicinal diol-containing molecules, thereby increasing local molecular organization at the membrane surface.

Although this model is hypothetical, it illustrates a chemically plausible mechanism by which reversible borate coordination could have generated adaptive, self-organizing protocellular membranes. Within the conceptual framework proposed in this review, borate-mediated supramolecular assembly provides a potential physicochemical bridge between simple amphiphilic membranes and increasingly sophisticated membrane architectures capable of supporting compartmentalization, proto-informational molecular networks, and the subsequent emergence of phosphate-based cellular systems.

## 10. Conclusions

The emergence of biological membranes represents one of the most fundamental transitions in the origin of life because compartmentalization enabled the concentration, protection, and organization of increasingly complex chemical systems. Although fatty acids have traditionally been regarded as the principal building blocks of primitive membranes, the evidence reviewed here suggests that low-molecular-weight polyols may have played a broader and previously underappreciated role during the earliest stages of membrane evolution.

Abiotic carbon chemistry is predicted to generate substantial quantities of ethylene glycol, glycerol, tetritols, and related polyols, providing an abundant reservoir of multifunctional molecules capable of participating in amphiphile formation. Their multiple hydroxyl groups facilitate esterification, etherification, hydrogen bonding, and reversible borate coordination, making them particularly suitable as hydrophilic scaffolds for increasingly complex proto-lipids. The widespread occurrence of polyol-containing membrane lipids in modern biology—including glycerolipids, glycolipids, sulfolipids, and archaeal ether lipids—demonstrates the remarkable versatility and long-term evolutionary success of polyol-based membrane architectures. These modern systems are viewed here as structural and functional analogues rather than direct evolutionary descendants of prebiotic membranes.

Within the conceptual framework proposed in this review, membrane evolution occurred in a chemically heterogeneous pre-phosphate Earth in which environmental pH acted as a major geochemical regulator of molecular selection, supramolecular organization, and membrane self-assembly. Sulfur-rich acidic environments may have favored sulfur-containing amphiphiles and dynamic sulfur-associated protomembranes; near-neutral environments likely promoted mixed polyol–fatty acid proto-lipids and increasingly stable membrane assemblies; and alkaline boron-rich systems selectively stabilized sugars and polyols through reversible borate complexation, facilitating the emergence of borate-associated amphiphiles and increasingly organized membrane structures. Rather than representing competing evolutionary scenarios, these complementary pH-dependent domains may have interacted continuously through geological and hydrological processes, collectively promoting membrane diversification and increasing molecular complexity.

An important feature of this model is the proposed role of hydrated polyol-rich networks and hydrogels as transitional physicochemical systems linking molecular synthesis with compartment formation. Borate-cross-linked polyol assemblies may have concentrated organic molecules, promoted amphiphilic self-assembly, stabilized reactive intermediates, and generated hydrated microenvironments favorable for increasingly organized chemical processes. Such dynamic soft-matter systems provide a plausible bridge between abiotic organic chemistry, protomembranes, and the emergence of proto-informational molecular networks.

This review further proposes that membrane evolution and informational organization were closely interconnected. Sulfur-associated molecular networks, borate-mediated stabilization of carbohydrates, and progressively more sophisticated membrane architectures may have acted cooperatively to establish the physicochemical conditions necessary for the emergence of increasingly organized prebiotic systems, ultimately facilitating the transition to phosphate-based RNA and modern cellular membranes. Although this evolutionary scenario remains hypothetical, it integrates established principles of geochemistry, supramolecular chemistry, membrane biophysics, and prebiotic chemistry into a coherent conceptual framework.

Future investigations should focus on experimentally evaluating the formation, stability, permeability, and self-assembly of polyol-derived amphiphiles under geochemically realistic prebiotic conditions, particularly across different pH regimes and in the presence of sulfur species and borate. Particular attention should be given to the properties of mixed amphiphilic systems, reversible borate coordination, hydrated polyol networks, and membrane-associated supramolecular organization. Such studies will help determine whether the pH-dependent evolutionary framework proposed here represents a plausible pathway toward protocellular membrane evolution.

Overall, the evidence reviewed here supports the hypothesis that low-molecular-weight polyols were not merely secondary products of prebiotic chemistry but may have served as fundamental structural building blocks during the emergence of proto-lipids and protomembranes on the pre-phosphate Earth. By integrating polyol chemistry, sulfur-rich geochemistry, borate-mediated molecular selection, hydrogel formation, amphiphilic self-assembly, and pH-dependent environmental organization into a unified evolutionary framework, this review offers a new perspective on one of the central questions in origin-of-life research. Most importantly, it presents a series of experimentally testable hypotheses that link geochemical environments with membrane evolution, providing a foundation for future investigations into the physicochemical origins of cellular compartmentalization and early biological organization.

## Figures and Tables

**Table 1:** Predicted distribution of prebiotic polyols.

Compound Class	Approx. Abundance
Ethylene glycol	40%
Glycerol	33%
Tetritols	17%
Pentitols	6%
Higher polyols	<4%

**Table 2:** Environmental Domains in the History of the Early Earth.

Environmental Domain	Dominant Chemistry	Preferred Molecular Systems
Acidic	Sulfur	Thiols, thioesters, sulfolipids, sulfur-rich carbohydrates
Neutral	Mixed	Fatty acids, glycolipids, amphiphiles, proto-lipids
Alkaline	Boron	Polyols, sugars, borate complexes, proto-informational assemblies
Transition Zones	Sulfur–Boron Interactions	Borate-stabilized amphiphiles, proto-membranes, emerging informational systems

**Table 3:** Functions of Phosphorus and Sulfur and Various Geological Periods of the Earth.

No	Modern Phosphate Role	Possible Sulfur-Era Analogue
1	Structural linkage	Sulfate esters
2	Energy transfer	Thioesters
3	Catalysis	Metal-sulfur clusters
4	Molecular recognition	Sulfated sugars
5	Membrane organization	Sulfolipids
6	Network regulation	Sulfur redox chemistry
7	Information transfer	Sulfur-rich compositional networks

**Table 4:** Environmental domains and Principal membrane types.

Environmental Domain	Approximate pH	Dominant Chemistry	Principal Membrane Type
Acidic	<6.5	Sulfur chemistry	Sulfo-protolipid membranes
Neutral	6.5–7.5	Fatty acids + polyols	Mixed polyol–fatty acid protomembranes
Alkaline	>8.0	Borate chemistry	Borate-associated protomembranes

**Table 5:** Comparative stability of various types of amphiphiles.

Property	Fatty Acid	Polyol Lipid	Sulfolipid	Borate Lipid
Stability	+	++	+++	+++
Hydrolysis	High	Moderate	Low	Moderate
Bilayer formation	Yes	Excellent	Excellent	Excellent
Hydrogen bonding	Low	High	High	Very high
pH tolerance	Narrow	Moderate	Acidic	Alkaline

## Data Availability

No new data were created or analyzed in this study. Data sharing is not applicable to this article.

## References

[1] Dembitsky V.M., Rosenberg G.S., Zanfera V.M. (2023). The Evolutionary Pathway to the Biomembrane: The Role of Low Molecular Weight Polyols in the Formation of the Protomembrane.

[2] Dembitsky V.M., Cambie R.Q. (1989). Ether lipids of the organic world: Formation and biotransformation. Fats for the Future.

[3] Deamer D. (2022). Origins of Life Research: The Conundrum between Laboratory and Field Simulations of Messy Environments. Life.

[4] Peretó J. (2019). Prebiotic chemistry that led to life. Handbook of Astrobiology.

[5] Gómez-Márquez J. (2025). The Origin of Life and Cellular Systems: A Continuum from Prebiotic Chemistry to Biodiversity. Life.

[6] Dembitsky V.M., Terent’ev A.O., Scorei I.R.I. (2026). Borate-Bridged Protolipids: A Prebiotic Route to Abiotic Membranes. Life.

[7] Deamer D. (2017). The Role of Lipid Membranes in Life’s Origin. Life.

[8] Solé R., Kempes C., Stepney S. (2025). Origins of Life: The Possible and the Actual. Philos. Trans. R. Soc. B Biol. Sci..

[9] Monnard P.A., Deamer D.W. (2010). Membrane Self-Assembly Processes: Steps toward the First Cellular Life. The Minimal Cell: The Biophysics of Cell Compartment and the Origin of Cell Functionality.

[10] Gözen I., Köksal E.S., Põldsalu I., Xue L., Spustova K., Pedrueza-Villalmanzo E., Ryskulov R., Meng F., Jesorka A. (2022). Protocells: Milestones and Recent Advances. Small.

[11] Serra R., Villani M. (2017). Modelling Protocells.

[12] Dzieciol A.J., Mann S. (2012). Designs for Life: Protocell Models in the Laboratory. Chem. Soc. Rev..

[13] Poka M.S., Milne M., Wessels A., Aucamp M. (2023). Sugars and Polyols of Natural Origin as Carriers for Solubility and Dissolution Enhancement. Pharmaceutics.

[14] Dembitsky V.M. (2025). Boronolipids. Vietnam J. Chem..

[15] Kurkowiak K., Emmerich L., Militz H. (2022). Wood Chemical Modification Based on Bio-Based Polycarboxylic Acid and Polyols—Status Quo and Future Perspectives. Wood Mater. Sci. Eng..

[16] de Souza F.M., Kahol P.K., Gupta R.K. (2021). Polyols from Sustainable Resources. Polyurethane Chemistry: Renewable Polyols and Isocyanates.

[17] Dembitsky V.M., Terent’ev A.O., Gursky M.E., Baranin S.V. (2026). Fascinating and Intriguing Biomolecules: The Chemistry of Boron Complexes with Carbohydrates, Glycolipids, and Steroids. Vietnam J. Chem..

[18] Pappin B., Kiefel M.J., Houston T.A. (2012). Boron–Carbohydrate Interactions. Carbohydrates—Comprehensive Studies on Glycobiology and Glycotechnology.

[19] Dembitsky V.M., Al Quntar A.A.A., Srebnik M. (2011). Natural and Synthetic Small Boron-Containing Molecules as Potential Inhibitors of Bacterial and Fungal Quorum Sensing. Chem. Rev..

[20] Scorei R., Cimpoiaşu V.M. (2006). Boron Enhances the Thermostability of Carbohydrates. Orig. Life Evol. Biosph..

[21] Dembitsky V.M., Smoum R., Al-Quntar A.A., Ali H.A., Pergament I., Srebnik M. (2002). Natural Occurrence of Boron-Containing Compounds in Plants, Algae and Microorganisms. Plant Sci..

[22] Dembitsky V.M., Terent’ev A.O., Baranin S.V., Gursky M.E. (2025). Aromatic Compounds and Their Fascinating Boron Complexes as Potential Quorum Sensing Molecules. Vietnam J. Chem..

[23] Dembitsky V.M., Terent’ev A.O., Stolbov L.A., Pogodin P.V., Filimonov D.A., Poroikov V.V. (2025). Salicylic Acid and Its Boron Complexes as Quorum Sensing Molecules. Mol. Pharm..

[24] Schopf J.W. (1982). Earth’s Earliest Biosphere: Its Origin and Evolution.

[25] Pilcher C.B. (2003). Biosignatures of Early Earths. Astrobiology.

[26] Levchenko V.F., Kazansky A.B., Sabirov M.A., Semenova E.M. (2012). Early Biosphere: Origin and Evolution.

[27] Fedonkin M.A. (2008). Ancient Biosphere: The Origin, Trends and Events. Russ. J. Earth Sci..

[28] Martin R.S., Mather T.A., Pyle D.M. (2007). Volcanic Emissions and the Early Earth Atmosphere. Geochim. Cosmochim. Acta.

[29] Polat A., Hofmann A.W., Rosing M.T. (2002). Boninite-Like Volcanic Rocks in the 3.7–3.8 Ga Isua Greenstone Belt, West Greenland: Geochemical Evidence for Intra-Oceanic Subduction Zone Processes in the Early Earth. Chem. Geol..

[30] Colman D.R., Lindsay M.R., Amenabar M.J., Fernandes-Martins M.C., Roden E.R., Boyd E.S. (2020). Phylogenomic Analysis of Novel Diaforarchaea Is Consistent with Sulfite but Not Sulfate Reduction in Volcanic Environments on Early Earth. ISME J..

[31] Pasek M.A. (2008). Rethinking Early Earth Phosphorus Geochemistry. Proc. Natl. Acad. Sci. USA.

[32] Walton C.R., Ewens S., Coates J.D., Blake R.E., Planavsky N.J., Reinhard C., Ju P., Hao J., Pasek M.A. (2023). Phosphorus Availability on the Early Earth and the Impacts of Life. Nat. Geosci..

[33] Ritson D.J., Mojzsis S.J., Sutherland J.D. (2020). Supply of Phosphate to Early Earth by Photogeochemistry after Meteoritic Weathering. Nat. Geosci..

[34] Farquhar J., Wing B.A., McKeegan K.D., Harris J.W., Cartigny P., Thiemens M.H. (2002). Mass-Independent Sulfur of Inclusions in Diamond and Sulfur Recycling on Early Earth. Science.

[35] Canfield D.E., Raiswell R. (1999). The Evolution of the Sulfur Cycle. Am. J. Sci..

[36] Ranjan S., Todd Z.R., Sutherland J.D., Sasselov D.D. (2018). Sulfidic Anion Concentrations on Early Earth for Surficial Origins-of-Life Chemistry. Astrobiology.

[37] Fakhraee M., Crockford P.W., Bauer K.W., Pasquier V., Sugiyama I., Katsev S., Planavsky N. (2025). The History of Earth’s Sulfur Cycle. Nat. Rev. Earth Environ..

[38] Canfield D.E., Habicht K.S., Thamdrup B. (2000). The Archean Sulfur Cycle and the Early History of Atmospheric Oxygen. Science.

[39] Domagal-Goldman S.D., Kasting J.F., Johnston D.T., Farquhar J. (2008). Organic Haze, Glaciations and Multiple Sulfur Isotopes in the Mid-Archean Era. Earth Planet. Sci. Lett..

[40] Kubota Y., Matsu’ura F., Shimizu K., Ishikawa A., Ueno Y. (2022). Sulfur in Archean Komatiite Implies Early Subduction of Oceanic Lithosphere. Earth Planet. Sci. Lett..

[41] Eguchi J., Black B.A., Nisbet E.G., Moussallam Y., Georgeais G. (2026). Secular Evolution of Mantle Sulfur Isotopes since the Archean. Geochem. Geophys. Geosyst..

[42] Kasting J.F., Zahnle K.J., Pinto J.P., Young A.T. (1989). Sulfur, Ultraviolet Radiation, and the Early Evolution of Life. Orig. Life Evol. Biosph..

[43] Wang B., Zuo R., Morrison S.M. (2025). Data-Driven Discovery of Sulfur Mineralogy. Math. Geosci..

[44] Lee J., Keum J., Kim T., Lee C., Chariton S., Prakapenka V., Giordano N., So B., Hwang H., Lee Y. (2026). Thermal Evolution of the Sulfur-Rich Small Terrestrial Planetary Core as Inferred from the Experimental Study of the Fe–S–O–H System. J. Geophys. Res. Planets.

[45] Helvacı C. (2017). Borate Deposits: An Overview and Future Forecast with Regard to Mineral Deposits. J. Boron.

[46] Bailey D.G., Lupulescu M.V., Darling R.S., Singer J.W., Chamberlain S.C. (2019). A Review of Boron-Bearing Minerals (Excluding Tourmaline) in the Adirondack Region of New York State. Minerals.

[47] Bulut G., Aydın Ş.B., Perek K.T., Arslan F. (2026). Enrichment of Boron Using Physical and Chemical Methods: A Review. Miner. Process. Extr. Metall. Rev..

[48] McClary C.A., Taylor M.S. (2013). Applications of Organoboron Compounds in Carbohydrate Chemistry and Glycobiology: Analysis, Separation, Protection, and Activation. Carbohydr. Res..

[49] van den Berg R., Peters J.A., van Bekkum H. (1995). Selective Alkaline Oxidative Degradation of Mono- and Di-Saccharides by Hydrogen Peroxide Using Borate as Catalyst and Protecting Group. Carbohydr. Res..

[50] Dembitsky V.M., Terent’ev A.O. (2026). Boron as a chemical editor: Dynamic selection of sugars in prebiotic environ-ments. Ptom. J. Chem..

[51] Weser U. (2008). Chemistry and Structure of Some Borate Polyol Compounds of Biochemical Interest. Structure and Bonding.

[52] Ricardo A., Carrigan M.A., Olcott A.N., Benner S.A. (2004). Borate Minerals Stabilize Ribose. Science.

[53] Furukawa Y., Horiuchi M., Kakegawa T. (2013). Selective Stabilization of Ribose by Borate. Orig. Life Evol. Biosph..

[54] Franco A., da Silva J.A.L. (2021). Boron in Prebiological Evolution. Angew. Chem. Int. Ed..

[55] Gulick A. (1955). Phosphorus as a Factor in the Origin of Life. Am. Sci..

[56] Walsh C.T. (2020). Chemical Biology of Phosphorus.

[57] Fernández-García C., Coggins A.J., Powner M.W. (2017). A Chemist’s Perspective on the Role of Phosphorus at the Origins of Life. Life.

[58] Pasek M. (2019). A Role for Phosphorus Redox in Emerging and Modern Biochemistry. Curr. Opin. Chem. Biol..

[59] Scorei R. (2012). Is Boron a Prebiotic Element? A Mini-Review of the Essentiality of Boron for the Appearance of Life on Earth. Orig. Life Evol. Biosph..

[60] Roy C., Mondal N., Peketi A., Fernandes S., Mapder T., Volvoikar S.P., Haldar P.K., Nandi N., Bhattacharya T., Mazumdar A. (2020). Geomicrobial Dynamics of the Trans-Himalayan Sulfur–Borax Spring System Reveals Mesophilic Bacteria’s Resilience to High Heat. J. Earth Syst. Sci..

[61] Mohamad H.M., Zakaria S.N.F., Sarjadi M.S., Musta B., Suro S.M. (2025). Investigation of Physicochemical Properties of Tabin Mud Volcano. Front. Water Environ..

[62] Brady M.P., Tostevin R., Tosca N.J. (2022). Marine Phosphate Availability and the Chemical Origins of Life on Earth. Nat. Commun..

[63] Atlas E., Pytkowicz R.M. (1977). Solubility Behavior of Apatites in Seawater. Limnol. Oceanogr..

[64] Berner R.A. (1973). Phosphate Removal from Sea Water by Adsorption on Volcanogenic Ferric Oxides. Earth Planet. Sci. Lett..

[65] Pratt A.J. (2006). The Curious Case of Phosphate Solubility. Chem. N. Z..

[66] Hao J., Li X., Ju P., Pasek M. (2025). Active Redox Cycling of Phosphorus on the Early Earth. Nat. Commun..

[67] Sutton S.M., Pulletikurti S., Lin H., Krishnamurthy R., Liotta C.L. (2025). Abiotic Aldol Reactions of Formaldehyde with Ketoses and Aldoses—Implications for the Prebiotic Synthesis of Sugars by the Formose Reaction. Chem.

[68] Touster O., Shaw D.R.D. (1962). Biochemistry of the Acyclic Polyols. Physiol. Rev..

[69] Bieleski R.L., Loewus F.A., Tanner W. (1982). Sugar Alcohols. Plant Carbohydrates I: Intracellular Carbohydrates.

[70] Cleaves H.J. (2011). A Hypothesis for a Unified Mechanism of Formation and Enantioenrichment of Polyols and Aldaric, Aldonic, Amino, Hydroxy and Sugar Acids in Carbonaceous Chondrites. Origins of Life: The Primal Self-Organization.

[71] Butlerow A.M. (1861). Carbohydrates Undergo an Aldol Condensation Reaction with Formaldehyde. Compt. Rend..

[72] Butlerow A. (1861). Bildung Einer Zuckerartigen Substanz Durch Synthese. Justus Liebigs Ann. Chem..

[73] Mizuno T., Weiss A.H. (1974). Synthesis and Utilization of Formose Sugars. Advances in Carbohydrate Chemistry and Biochemistry.

[74] Kopetzki D., Antonietti M. (2011). Hydrothermal Formose Reaction. New J. Chem..

[75] Weiss A.H., Socha R.F., Likholobov V.A., Sakharov M.M. (1981). Formose Sugars from Formaldehyde. Appl. Catal..

[76] Omran A., Menor-Salvan C., Springsteen G., Pasek M. (2020). The Messy Alkaline Formose Reaction and Its Link to Metabolism. Life.

[77] Zhao Y., Biggs T.D., Xian M. (2014). Hydrogen Sulfide (H2S)-Releasing Agents: Chemistry and Biological Applications. Chem. Commun..

[78] Carter D.E. (1995). Oxidation–Reduction Reactions of Metal Ions. Environ. Health Perspect..

[79] Buerge I.J., Hug S.J. (1999). Influence of Mineral Surfaces on Chromium(VI) Reduction by Iron(II). Environ. Sci. Technol..

[80] Makkee M., Kieboom A.P.G., van Bekkum H. (1985). Studies on Borate Esters III. Borate Esters of D-Mannitol, D-Glucitol, D-Fructose and D-Glucose in Water. Recl. Trav. Chim. Pays-Bas.

[81] Coddington J.M., Taylor M.J. (1989). High-Field11B and ^13^C NMR Investigations of Aqueous Borate Solutions and Borate–Diol Complexes. J. Coord. Chem..

[82] Henderson W.G., How M.J., Kennedy G.R., Mooney E.F. (1973). The Interconversion of Aqueous Boron Species and the Interaction of Borate with Diols: A ^11^B NMR Study. Carbohydr. Res..

[83] Maseda M., Miyazaki Y., Takamuku T. (2021). Thermodynamics for Complex Formation of Boric Acid and Borate with Hydroxy Acids and Diols. J. Mol. Liq..

[84] Šponer J.E., Sumpter B.G., Leszczynski J., Šponer J., Fuentes-Cabrera M. (2008). Theoretical Study on the Factors Controlling the Stability of the Borate Complexes of Ribose, Arabinose, Lyxose, and Xylose. Chem. Eur. J..

[85] Umesh R., Harohally N.V. (2025). Geochemical Settings at an Alkaline Hydrothermal Vent Stabilized Ribose: Evidence from Chemical Garden and Other Simulation Experiments. ACS Earth Space Chem..

[86] Takahashi Y., Kim H.J., Benner S.A., Kakegawa T., Furukawa Y. (2026). Ribose Accumulation in Borate-Rich Prebiotic Environments. Astrobiology.

[87] Scorei I.R. (2025). Boron and the Three Evolutionary Stages of Life from Chemical Emergence to Digital Continuity. Discov. Life.

[88] Dembitsky V.M., Terent’ev A.O. (2026). Boron–Vicinal Diol Xanthophyll Complexes as Emerging Photoprotective Adjuvants. Photochem.

[89] Koonin E.V. (2014). The origins of cellular life. Antonie Leeuwenhoek.

[90] Vázquez-Salazar A. (2026). Complex and Messy Prebiotic Chemistry: Obstacles and Opportunities for an RNA World. Life.

[91] Tang W., Shi X., Shen J., Mao H., Wei E., Huang B., Ying J., Cai H., Zhao Y. (2026). Metal-Ion Mediated Photolytic Regulation of Diphenylalanine Stability: Implications for Prebiotic Chemical Evolution. iScience.

[92] Marlin T.C., Weber J.M., Sheppard R.Y., Perl S., Diener D., Baum M.M., Barge L.M. (2025). Chemical Gardens as Analogs for Prebiotic Chemistry on Ocean Worlds. Chem.

[93] Brimblecombe P., Norman A.L. (2026). Sulfur in the Earth’s atmosphere. The Role of Sulfur in Planetary Processes: From Cores to Atmospheres.

[94] Amenabar M.J., Boyd E.S. (2019). A Review of the Mechanisms of Mineral-Based Metabolism in Early Earth Analog Rock-Hosted Hydrothermal Ecosystems. World J. Microbiol. Biotechnol..

[95] Halevy I., Schrag D.P. (2009). Sulfur Dioxide Inhibits Calcium Carbonate Precipitation: Implications for Early Mars and Earth. Geophys. Res. Lett..

[96] Ren X.Y., Lin X.Y., Tanabe T.S., Loy A., Hu L., Zhu Y.G., Chen S.C. (2026). Evolutionary trajectory of micro-bial sulfur oxidation pathways recapitulates Earth’s oxygenation history. Nat. Commun..

[97] Vignale F.A., Sánchez-García L., Carrizo D., Sepúlveda A.C., Taubner H., Oriolo S., Mitchell A.L., Turjanski A.G., Klatt J.M., Finn R.D. (2025). Seasonal Biogeochemical Variations in a Modern Microbialite Reef under Early Earth-Like Conditions. Commun. Earth Environ..

[98] Matreux T., Schmid A., Rappold M., Weller D., Çalışkanoğlu A.Z., Moore K.R., Bosak T., Dingwell D.B., Karaghiosoff K., Guyot F. (2025). Heat Flows Solubilize Apatite to Boost Phosphate Availability for Prebiotic Chemistry. Nat. Commun..

[99] Krissansen-Totton J., Arney G.N., Catling D.C. (2018). Constraining the Climate and Ocean pH of the Early Earth with a Geological Carbon Cycle Model. Proc. Natl. Acad. Sci. USA.

[100] Toner J.D., Catling D.C. (2019). Alkaline Lake Settings for Concentrated Prebiotic Cyanide and the Origin of Life. Geochim. Cosmochim. Acta.

[101] Kadoya S., Krissansen-Totton J., Catling D.C. (2020). Probable Cold and Alkaline Surface Environment of the Hadean Earth Caused by Impact Ejecta Weathering. Geochem. Geophys. Geosyst..

[102] Schiedung M., Harrington K.J., Dupla X., Möller B., Facq E., Sweere T., Don A., Hilton R.G., Doetterl S., Hemingway J.D. (2026). Uncertainties of Enhanced Rock Weathering for Climate-Change Mitigation. Nat. Rev. Earth Environ..

[103] Arndt N.T., Nisbet E.G. (2012). Processes on the Young Earth and the Habitats of Early Life. Annu. Rev. Earth Planet. Sci..

[104] Halevy I., Bachan A. (2017). The Geologic History of Seawater pH. Science.

[105] Lopalco A., Lopedota A.A., Laquintana V., Denora N., Stella V.J. (2020). Boric Acid, a Lewis Acid with Unique and Unusual Properties: Formulation Implications. J. Pharm. Sci..

[106] Zumreoglu-Karan B., Kose D.A. (2015). Boric Acid: A Simple Molecule of Physiologic, Therapeutic and Prebiotic Significance. Pure Appl. Chem..

[107] Sojo V., Herschy B., Whicher A., Camprubi E., Lane N. (2016). The Origin of Life in Alkaline Hydrothermal Vents. Astrobiology.

[108] Kitadai N., Nakamura R., Yamamoto M., Takai K., Yoshida N., Oono Y. (2019). Metals Likely Promoted Protometabolism in Early Ocean Alkaline Hydrothermal Systems. Sci. Adv..

[109] Herschy B., Whicher A., Camprubi E., Watson C., Dartnell L., Ward J., Evans J.R.G., Lane N. (2014). An Origin-of-Life Reactor to Simulate Alkaline Hydrothermal Vents. J. Mol. Evol..

[110] Benner S.A. (2004). Understanding Nucleic Acids Using Synthetic Chemistry. Acc. Chem. Res..

[111] Knoeck J., Taylor J.K. (1969). Aqueous Boric Acid–Borate–Mannitol Equilibria. Anal. Chem..

[112] Alexzman Z.A., Ibrahim M.L., Annuar N.H.R. (2023). A Short Review on Catalytic Hydrogenation of Fructose into Mannitol. J. Chem. Technol. Biotechnol..

[113] Konings W.N., Albers S.V., Koning S., Driessen A.J.M. (2002). The Cell Membrane Plays a Crucial Role in Survival of Bacteria and Archaea in Extreme Environments. Antonie van Leeuwenhoek.

[114] Gould S.B. (2018). Membranes and Evolution. Curr. Biol..

[115] van Wolferen M., Pulschen A.A., Baum B., Gribaldo S., Albers S.V. (2022). The Cell Biology of Archaea. Nat. Microbiol..

[116] Kates M. (1993). Membrane Lipids of Archaea. New Comprehensive Biochemistry.

[117] Ulrih N.P., Gmajner D., Raspor P. (2009). Structural and Physicochemical Properties of Polar Lipids from Thermophilic Archaea. Appl. Microbiol. Biotechnol..

[118] Zeng Z., Liu X.L., Wei J.H., Summons R.E., Welander P.V. (2018). Calditol-Linked Membrane Lipids Are Required for Acid Tolerance in *Sulfolobus acidocaldarius*. Proc. Natl. Acad. Sci. USA.

[119] Koga Y., Morii H. (2005). Recent Advances in Structural Research on Ether Lipids from Archaea Including Comparative and Physiological Aspects. Biosci. Biotechnol. Biochem..

[120] Stonik V.A., Makarieva T.N., Shubina L.K., Guzii A.G., Ivanchina N.V. (2024). Structure Diversity and Properties of Some Bola-Like Natural Products. Mar. Drugs.

[121] Li Y., Ma L., Huang S., Chen S., Begum S., Ibrahim N., Liang Y., Liu X. (2025). Roles of Mobile Genetic Elements and Biosynthetic Gene Clusters in Environmental Adaptation of Acidophilic Archaeon *Ferroplasma* to Extreme Polluted Environments. Front. Microbiol..

[122] Jala R.C.R., Vudhgiri S., Kumar C.G. (2022). A Comprehensive Review on Natural Occurrence, Synthesis and Biological Activities of Glycolipids. Carbohydr. Res..

[123] Curatolo W. (1987). The Physical Properties of Glycolipids. Biochim. Biophys. Acta Rev. Biomembr..

[124] Goodby J.W., Görtz V., Cowling S.J., Mackenzie G., Martin P., Plusquellec D., Benvegnu T., Boullanger P., Lafont D., Queneau Y. (2007). Thermotropic Liquid Crystalline Glycolipids. Chem. Soc. Rev..

[125] Kim E., Kobayashi K. (2026). Membrane Lipid Properties in Photosynthetic Responses to Environmental Changes. J. Exp. Bot..

[126] Harwood J.L. (1980). Sulfolipids. Lipids: Structure and Function.

[127] Shimojima M. (2011). Biosynthesis and Functions of the Plant Sulfolipid. Prog. Lipid Res..

[128] Haines T.H. (1973). Sulfolipids and Halosulfolipids. Lipids and Biomembranes of Eukaryotic Microorganisms.

[129] Martín-Alfonso M.J., Martínez-Boza F.J., Luckham P.F. (2025). Bio-Based Guar–Borate Hydrogels: Processing Effects and Rheological Insights for High-Temperature Applications. J. Polym. Environ..

[130] Peters G.M., Skala L.P., Plank T.N., Oh H., Reddy G.N.M., Marsh A., Brown S.P., Raghavan S.R., Davis J.T. (2015). G4-Quartet·M+ Borate Hydrogels. J. Am. Chem. Soc..

[131] Guan Y., Zhang Y. (2013). Boronic Acid-Containing Hydrogels: Synthesis and Their Applications. Chem. Soc. Rev..

[132] Yu Y., Zhang L., Hu B., Wang Z., Gu Q., Wang W., Zhu C., Wang S. (2024). Borate Bonds-Containing pH-Responsive Chitosan Hydrogel for Postoperative Tumor Recurrence and Wound Infection Prevention. Carbohydr. Polym..

[133] Wu H., Su Q., Zhou R., Yan J., Feng Y., Guan C., Su W., Chen D., Tang L., Huang X. (2025). Synergistic Borate Crosslinking and Chain Entanglement for Mechanically Robust and Water-Rich Hydrogels. J. Colloid Interface Sci..

[134] Peppas N.A., Hoffman A.S. (2020). Hydrogels. Biomaterials Science.

[135] Zhang Y.S., Khademhosseini A. (2017). Advances in Engineering Hydrogels. Science.

[136] Chirani N., Yahia L.H., Gritsch L., Motta F.L., Chirani S., Farè S. (2015). History and Applications of Hydrogels. J. Biomed. Sci..

[137] Yang D. (2022). Recent Advances in Hydrogels. Chem. Mater..

[138] Morasch M., Liu J., Dirscherl C.F., Ianeselli A., Kühnlein A., Le Vay K., Schwintek P., Islam S., Corpinot M.K., Scheu B. (2019). Heated Gas Bubbles Enrich, Crystallize, Dry, Phosphorylate and Encapsulate Prebiotic Molecules. Nat. Chem..

[139] Khanum R., Satthiyasilan N., Tharumen N., Kee T.P., Mayer C., Menon P.S., Jia T.Z., Chandru K. (2026). Prebiotic Gels as the Cradle of Life. ChemSystemsChem.

[140] Trevors J.T. (2011). Hypothesized Origin of Microbial Life in a Prebiotic Gel and the Transition to a Living Biofilm and Microbial Mats. Comptes Rendus Biol..

[141] Nogal N., Sanz-Sánchez M., Vela-Gallego S., Ruiz-Mirazo K., de la Escosura A. (2023). The Protometabolic Nature of Prebiotic Chemistry. Chem. Soc. Rev..

[142] Yang D., Peng S., Hartman M.R., Gupton-Campolongo T., Rice E.J., Chang A.K., Gu Z., Lu G.Q., Luo D. (2013). Enhanced Transcription and Translation in Clay Hydrogel and Implications for Early Life Evolution. Sci. Rep..

[143] Li B., Liu J., Lyu F., Deng Z., Yi B., Du P., Yao X., Zhu G., Xu Z., Lu J. (2022). Mineral Hydrogel from Inorganic Salts: Biocompatible Synthesis, All-in-One Charge Storage, and Possible Implications in the Origin of Life. Adv. Funct. Mater..

[144] Sarkar S., Das S., Dagar S., Joshi M.P., Mungi C.V., Sawant A.A., Patki G.M., Rajamani S. (2020). Prebiological Membranes and Their Role in the Emergence of Early Cellular Life. J. Membr. Biol..

[145] D’Almeida A.P., Infante-Neta A.A., Arcanjo M.R.A., Albuquerque T.L.D. (2026). Probiotics and Prebiotics in Dairy: Enhancing Health, Quality, and Sensorial Properties. Fermentation.

[146] Shvetsova A., Fiore M. (2026). Chemical Routes to Primitive Membranes: Prebiotic Lipid Formation at the Origin of Life. Life.

[147] Massoth M. (2026). From Physical Difference to Meaning: A Constructor-Theoretic Framework for Prebiotic Information in Casimir–Lifshitz-Coupled Protocell Clusters. arXiv.

[148] Chakraborty P. (2025). Spontaneous Structured Intelligence via Biphasic Interfacial Redox-Chelation: Chakraborty’s Redox World Model for Prebiotic Non-Enzymatic Energy Cycle. ChemRxiv.

[149] Olson K.R., Straub K.D. (2016). The Role of Hydrogen Sulfide in Evolution and the Evolution of Hydrogen Sulfide in Metabolism and Signaling. Physiology.

[150] Rickard D., Mussmann M., Steadman J.A. (2017). Sedimentary Sulfides. Elements.

[151] Casas A.S., King P.L., Delmelle P. (2026). Sulfur in volcanism. The Role of Sulfur in Planetary Processes: From Cores to Atmospheres.

[152] Bradley A.S. (2026). Biogeochemistry of Sulfur. The Role of Sulfur in Planetary Processes: From Cores to Atmospheres.

[153] Neveu M., Kim H.J., Benner S.A. (2013). The “Strong” RNA World Hypothesis: Fifty Years Old. Astrobiology.

[154] Benner S.A., Kim H.J., Yang Z. (2012). Setting the Stage: The History, Chemistry, and Geobiology behind RNA. Cold Spring Harb. Perspect. Biol..

[155] Benner S.A., Ellington A.D., Tauer A. (1989). Modern Metabolism as a Palimpsest of the RNA World. Proc. Natl. Acad. Sci. USA.

[156] Todd Z.R. (2022). Sources of Nitrogen-, Sulfur-, and Phosphorus-Containing Feedstocks for Prebiotic Chemistry in the Planetary Environment. Life.

[157] Li Y., Kitadai N., Nakamura R. (2018). Chemical Diversity of Metal Sulfide Minerals and Its Implications for the Origin of Life. Life.

[158] Wächtershäuser G. (1998). Origin of Life in an Iron–Sulfur World. The Molecular Origins of Life.

[159] Goldford J.E., Hartman H., Marsland R., Segrè D. (2019). Environmental Boundary Conditions for the Origin of Life Converge to an Organo-Sulfur Metabolism. Nat. Ecol. Evol..

[160] Nes W.R. (2012). Lipids in Evolution.

[161] Goddard-Borger E.D., Williams S.J. (2017). Sulfoquinovose in the Biosphere: Occurrence, Metabolism and Functions. Biochem. J..

[162] Hand K.P., Chyba C.F., Priscu J.C., Carlson R.W., Nealson K.H. (2009). Astrobiology and the Potential for Life on Europa. Europa.

[163] Itoh Y.H., Sugai A., Uda I., Itoh T. (2001). The Evolution of Lipids. Adv. Space Res..

[164] Jacques J., Maryse A.B., Agnes M., Eric M., Roland D. (2018). Origin and Synthesis of Galactolipid and Sulfolipid Head Groups. Lipid Metabolism in Plants.

[165] Vilanova E., Santos G.R., Aquino R.S., Valle-Delgado J.J., Anselmetti D., Fernandez-Busquets X., Mourão P.A.S. (2016). Carbohydrate–Carbohydrate Interactions Mediated by Sulfate Esters and Calcium Provide the Cell Adhesion Required for the Emergence of Early Metazoans. J. Biol. Chem..

[166] Devínsky F. (2021). Chirality and the Origin of Life. Symmetry.

[167] Andrulis E.D. (2012). Theory of the Origin, Evolution, and Nature of Life. Life.

[168] Hooper L.V., Manzella S.M., Baenziger J.U. (1997). The Biology of Sulfated Oligosaccharides. Glycosciences: Status and Perspectives.

[169] de Duve C. (1995). The Beginnings of Life on Earth. Am. Sci..

[170] de Duve C. (1998). Clues from Present-Day Biology: The Thioester World. The Molecular Origins of Life.

[171] Jakubowski H. (2017). Homocysteine Editing, Thioester Chemistry, Coenzyme A, and the Origin of Coded Peptide Synthesis. Life.

[172] Hill J. (2000). Sulfur and the Origins of Life. Master’s Thesis.

[173] Weber A.L. (1984). Prebiotic Formation of Energy-Rich Thioesters from Glyceraldehyde and *N*-Acetylcysteine. Orig. Life Evol. Biosph..

[174] Kuila S., Nanda J. (2024). Cysteine-Based Dynamic Self-Assembly and Their Importance in the Origins of Life. ChemSystemsChem.

[175] Ofosu D.B. (2024). Brimstone Life: The Role of Sulfur in the Origin of Life and Related Practical Applications. Ph.D. Thesis.

[176] Vallée Y., Shalayel I., Ly K.D., Rao K.R., de Paëpe G., Märker K., Milet A. (2017). At the Very Beginning of Life on Earth: The Thiol-Rich Peptide (TRP) World Hypothesis. Int. J. Dev. Biol..

[177] Siqueira-Batista R., Alves-Ferreira R., Maroca-de-Avelar R.L.P., Mayrink M.I.C.B., Silva E., Gómez F. (2026). Prebiotic Chemistry and Origin of Living Beings: Current State, Scientific Perspectives and (Bio)Ethical Issues. Front. Astron. Space Sci..

[178] Mescall K.A. (1994). The Chemical Origin of Life: An Exploration of Thioesters as Energy in the Prebiotic World. Ph.D. Thesis.

[179] Cech T.R. (2012). The RNA Worlds in Context. Cold Spring Harb. Perspect. Biol..

[180] Flores-García A.A., Hernandez-Rodriguez A., López-Brito E.I., Nava-Domínguez D.F., Pasapera S.P., Pazos-Murillo R.F., Pineda-Diaz T., Vázquez-Salazar A. (2026). Brave New RNA World(s): From Prebiotic Chemistry to Gene Regulation and RNA Technology. Front. Genet..

[181] Vitas M., Dobovišek A. (2025). A Possible Origin of Life in Nonpolar Environments. BioSystems.

[182] Michaelian K. (2022). Non-Equilibrium Thermodynamic Foundations of the Origin of Life. Foundations.

[183] Westall F., Brack A., Fairén A.G., Schulte M.D. (2023). Setting the Geological Scene for the Origin of Life and Continuing Open Questions about Its Emergence. Front. Astron. Space Sci..

[184] Ono C., Sunami S., Ishii Y., Kim H.J., Kakegawa T., Benner S.A., Furukawa Y. (2024). Abiotic Ribose Synthesis under Aqueous Environments with Various Chemical Conditions. Astrobiology.

[185] Gull M., Pasek M.A. (2021). The Role of Glycerol and Its Derivatives in the Biochemistry of Living Organisms, and Their Prebiotic Origin and Significance in the Evolution of Life. Catalysts.

[186] Simoneit B.R.T., Rushdi A.I., Deamer D.W. (2007). Abiotic Formation of Acylglycerols under Simulated Hydrothermal Conditions and Self-Assembly Properties of Such Lipid Products. Adv. Space Res..

[187] Hanczyc M.M., Monnard P.A. (2016). The Origin of Life and the Potential Role of Soaps. Lipid Technol..

[188] Segré D., Ben-Eli D., Deamer D.W., Lancet D. (2001). The Lipid World. Orig. Life Evol. Biosph..

[189] Oró J. (1995). Chemical Synthesis of Lipids and the Origin of Life. J. Biol. Phys..

[190] Dembitsky V.M., Terent’ev A.O. (2026). Boron–Diol Chemistry in Marine Systems: Biological Interfaces, Microbial Communication, and Therapeutic Potential. MarineMedicine.

[191] Luisi P.L., Walde P., Oberholzer T. (1999). Lipid Vesicles as Possible Intermediates in the Origin of Life. Curr. Opin. Colloid Interface Sci..

[192] Řezanka T., Kyselová L., Murphy D.J. (2023). Archaeal Lipids. Prog. Lipid Res..

[193] Rattanasriampaipong R., Zhang Y.G., Pearson A., Hedlund B.P., Zhang S. (2022). Archaeal Lipids Trace Ecology and Evolution of Marine Ammonia-Oxidizing Archaea. Proc. Natl. Acad. Sci. USA.

[194] Liman G.L.S., Garcia A.A., Fluke K.A., Anderson H.R., Davidson S.C., Welander P.V., Santangelo T.J. (2024). Tetraether Archaeal Lipids Promote Long-Term Survival in Extreme Conditions. Mol. Microbiol..

[195] Zhang J., Li T., Hong Z., Ma C., Fang X., Zheng F., Teng W., Zhang C., Si T. (2023). Biosynthesis of Hybrid Neutral Lipids with Archaeal and Eukaryotic Characteristics in Engineered *Saccharomyces cerevisiae*. Angew. Chem. Int. Ed..

[196] Ahangarian N., Umoh U.U., MacAdam N., MacDonald A., Granados P., Bentley J.N., Redshaw E., Fowler M.G., Baghalabadi V., Ventura G.T. (2026). Archaeal Lipostratigraphy of the Scotian Slope Shallow Sediments, Atlantic Canada. Biogeosciences.

[197] Kimiran A., Çağ M.M., Doğruöz Güngör N. (2026). Diversity of Archaea. Microbial World: Bacteria and Archaea.

[198] Cezanne A., Drobnič T., Fiege K., Kuo Y.-W., Parham J., Bale N.J., Fooa S., Löweb J., Villanueva L., Baum B. (2026). Bridging the Lipid Divide: Archaeal ESCRT-III Binds Phosphoinositol and Polarises the Cytokinetic Membrane. bioRxiv.

[199] Pekdemir A.D., Önal M., Sarikaya Y. (2023). Optimum pyrolysis conditions to prepare the most crystalline bo-ron carbide powder from boric acid–mannitol complex ester. Turk. J. Chem..

[200] Davis H.B., Mott C.J. (1980). Interaction of Boric Acid and Borates with Carbohydrates and Related Substances. J. Chem. Soc. Faraday Trans. 1.

[201] Nickerson R.F. (1968). The Combining Ratio of Boric Acid and Alkali Borate with Mannitol. J. Inorg. Nucl. Chem..

[202] Scorei I.R., Biţă A., Mogoşanu G.D., Hunter J.M. (2026). Calcium Fructoborate: Unique among Other Boron–Carbohydrate Complexes from Nature: Biochemical Roles and Pharmacological Potential. Handbook of Experimental Pharmacology.

[203] Acar Can S., Harman B.I., Koseoglu H. (2025). Enhanced Boron Removal with Polyols from Geothermal Waters by Ceramic Membrane. Int. J. Environ. Sci. Technol..

[204] Dembitsky V.M., Baranin S.V., Terent’ev A.O. (2025). 1,2-Diol Xanthophylls and Their Boron Complexes. MarineMedicine.

